# Para-Aminobenzoic Acid, Calcium, and c-di-GMP Induce Formation of Cohesive, Syp-Polysaccharide-Dependent Biofilms in Vibrio fischeri

**DOI:** 10.1128/mBio.02034-21

**Published:** 2021-10-05

**Authors:** Courtney N. Dial, Lauren Speare, Garrett C. Sharpe, Scott M. Gifford, Alecia N. Septer, Karen L. Visick

**Affiliations:** a Department of Microbiology and Immunology, Loyola University Chicago, Maywood, Illinois, USA; b Department of Earth, Marine and Environmental Sciences, The University of North Carolina at Chapel Hill, Chapel Hill, North Carolina, USA; c Environment, Ecology and Energy Program, The University of North Carolina at Chapel Hill, Chapel Hill, North Carolina, USA; University of Washington

**Keywords:** *Vibrio fischeri*, biofilms, c-di-GMP, calcium signaling, cyclic nucleotides, pABA, signal transduction

## Abstract

The marine bacterium Vibrio fischeri efficiently colonizes its symbiotic squid host, Euprymna scolopes, by producing a transient biofilm dependent on the symbiosis polysaccharide (SYP). *In vitro*, however, wild-type strain ES114 fails to form SYP-dependent biofilms. Instead, genetically engineered strains, such as those lacking the negative regulator BinK, have been developed to study this phenomenon. Historically, V. fischeri has been grown using LBS, a complex medium containing tryptone and yeast extract; supplementation with calcium is required to induce biofilm formation by a *binK* mutant. Here, through our discovery that yeast extract inhibits biofilm formation, we uncover signals and underlying mechanisms that control V. fischeri biofilm formation. In contrast to its inability to form a biofilm on unsupplemented LBS, a *binK* mutant formed cohesive, SYP-dependent colony biofilms on tTBS, modified LBS that lacks yeast extract. Moreover, wild-type strain ES114 became proficient to form cohesive, SYP-dependent biofilms when grown in tTBS supplemented with both calcium and the vitamin para-aminobenzoic acid (pABA); neither molecule alone was sufficient, indicating that this phenotype relies on coordinating two cues. pABA/calcium supplementation also inhibited bacterial motility. Consistent with these phenotypes, cells grown in tTBS with pABA/calcium were enriched in transcripts for biofilm-related genes and predicted diguanylate cyclases, which produce the second messenger cyclic-di-GMP (c-di-GMP). They also exhibited elevated levels of c-di-GMP, which was required for the observed phenotypes, as phosphodiesterase overproduction abrogated biofilm formation and partially rescued motility. This work thus provides insight into conditions, signals, and processes that promote biofilm formation by V. fischeri.

## INTRODUCTION

Bacteria recognize and respond to various signals in their environment and adapt accordingly. These signals can include temperature, pH, nutrients, small molecules, and even surfaces. These cues provide bacteria with important information with respect to their location and can promote behaviors necessary for bacteria to survive or thrive within their current environment. One such environment-induced behavior is the formation of a biofilm, or community of microorganisms connected by a protective matrix of secreted polysaccharides, proteins, and other molecules. A variety of signals are known to promote biofilm formation, including glucose sugars, amino acids, polyamines, and calcium (1).

The marine bacterium Vibrio fischeri serves as an important model for understanding the signals that control biofilm formation because this microbe naturally relies on this trait for successful colonization of its symbiotic host, the Hawaiian bobtail squid Euprymna scolopes (2, 3). The earliest interactions between these organisms result in the bacteria forming a biofilm on the surface of the symbiotic organ from which they disperse to enter and colonize the internal spaces of the organ (4). Symbiotic biofilm formation depends on the production of Syp polysaccharide (SYP) by proteins encoded by a conserved *syp* locus present in most *Vibrio* species, including the pathogens Vibrio vulnificus and Vibrio parahaemolyticus (5). V. fischeri mutants that fail to produce SYP exhibit colonization defects, while strains that overproduce Syp-dependent biofilms exhibit increased symbiotic aggregates and outcompete the wild-type strain for colonization (5–9).

In standard laboratory culture—typically a tryptone- and yeast extract-containing medium such as LBS (10)—SYP production is undetectable in the context of the canonical wild-type strain of V. fischeri ES114 (5, 7). Thus, the study of Syp-dependent biofilms has depended on the use of genetically modified strains with enhanced SYP production, achieved by overproducing positive regulators, such as RscS or SypG, and/or deleting genes for negative regulators, such as *binK* (6, 8, 9). Work reliant on such strains has revealed a complex network of regulators and other factors that contribute to production of cohesive, Syp-dependent biofilms (11–15). It has also permitted the identification of signals that impact biofilm formation (11). For example, calcium is a key trigger for inducing biofilm formation, causing a Δ*binK* mutant to switch from producing a smooth, nonsticky colony to one with cohesion and wrinkled architecture (11).

Furthermore, addition of calcium to shaking liquid LBS cultures of a Δ*binK* mutant induced it to produce not only SYP but also a second polysaccharide, cellulose (11, 16). The two calcium-induced biofilms could be distinguished by their relative cohesion or “stickiness”: SYP-dependent biofilms took the form of robustly cohesive clumps at the bottom of the test tubes of shaking cultures that were not readily disrupted by shaking or vortexing, while cellulose-dependent biofilms generally were visualized as a ring around the test tube in the “splash zone”; when cellulose-dependent clumps form (for example, with mutation of *syp*), they are readily dispersed by vortexing. The biofilms formed by the two polysaccharides combined to produce “trees” that connect the cellulose-dependent rings to the SYP-dependent clumps (11). Finally, and in contrast to SYP, cellulose production could be induced by calcium addition in wild-type strain ES114, which formed cellulose-dependent rings but not clumps or trees when grown with shaking in LBS containing calcium (11).

Biofilms are often promoted by cyclic-di-GMP (c-di-GMP), a ubiquitous second messenger in bacteria (17–20). High levels of c-di-GMP induce a sessile biofilm state, and low levels of c-di-GMP promote motility and/or switch cells to a planktonic state. c-di-GMP is generated by diguanylate cyclases (DGCs) and degraded by phosphodiesterases (PDEs). While cellulose production is known to be activated by c-di-GMP (16, 18, 21), a role for this small molecule in promoting SYP production has not been reported.

Previously, we described different media conditions that resulted in altered SYP-dependent colony biofilm phenotypes (distinct colony architecture, increased/decreased colony cohesiveness/adhesiveness, earlier/later timing of biofilm formation, etc.) (22). For example, an RscS-overproducing strain formed wrinkled colonies that were tightly adherent to the agar surface when grown on modified LBS medium that lacked yeast extract. In contrast, it formed more cohesive, less wrinkled colonies on modified LBS medium that lacked tryptone. These findings suggested that, in addition to calcium, other nutrients present in tryptone and/or yeast extract may control Syp-dependent biofilm formation.

Here, through the use of modified culture media, we uncovered signals and underlying mechanisms that control V. fischeri biofilm formation. Specifically, we determined that yeast extract inhibits biofilm formation by V. fischeri. By using a medium that lacks yeast extract, we identified calcium and the vitamin para-aminobenzoic acid (pABA) as coordinate positive inducers of cohesive SYP-dependent biofilm formation by wild-type strain ES114. These conditions thus overcome the need for genetically engineered strains. Furthermore, we determined that pABA and calcium induce numerous changes in the transcriptome and substantially increase levels of c-di-GMP, which was necessary for SYP-dependent biofilm formation by the wild-type strain. Together, these data provide new insights into the natural mechanisms governing V. fischeri biofilm formation.

## RESULTS

### Yeast extract and pABA inhibit biofilm formation.

**FIG 1:**
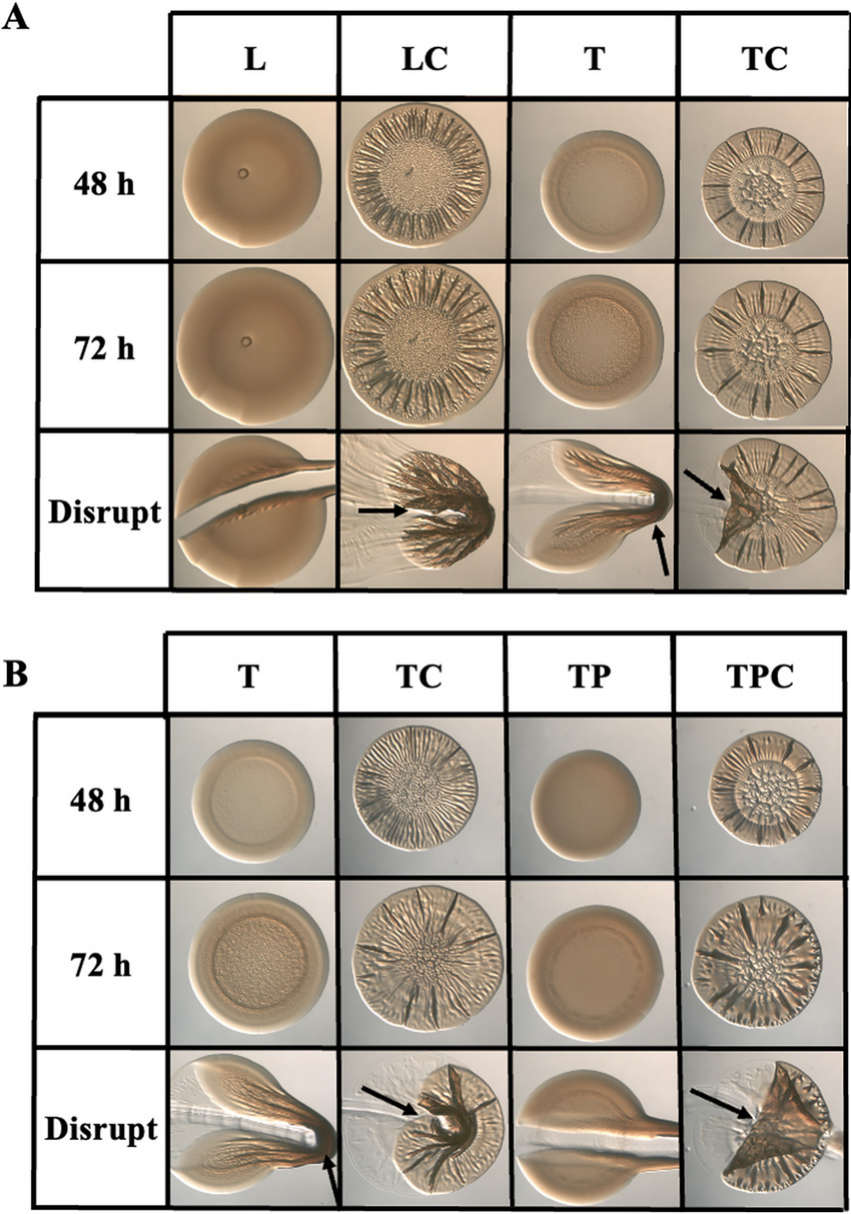
Yeast extract inhibits Δ*binK* mutant biofilm formation. (A) Colony biofilm formation by the Δ*binK* mutant (KV7860) was evaluated following growth on LBS (L), LBS + 10 mM calcium (LC), tTBS (T), and tTBS + 10 mM calcium (TC). (B) Colony biofilm formation by the Δ*binK* mutant (KV7860) was evaluated following growth on tTBS (T), tTBS + 10 mM calcium (TC), tTBS + 9.7 mM pABA (TP), and tTBS + pABA/calcium (TPC). Pictures were taken using a dissecting light microscope at 48 and 72 h. Each colony was disrupted using a toothpick after 72 h. Pictures are representative of 3 separate experiments. Arrows indicate where “pulling,” indicating cohesion, was observed.

In the course of experiments designed to evaluate the impact of media conditions on biofilm formation, we observed that yeast extract was inhibitory. As seen previously, a Δ*binK* mutant failed to form wrinkled colonies on the yeast-extract-containing medium LBS unless it was supplemented with calcium (Fig. 1A) (11). However, when grown in tTBS, modified LBS medium that lacks yeast extract, the Δ*binK* mutant produced cohesive colony biofilms even without calcium supplementation. We observed similar results on medium that contained tTBS with twice as much tryptone (2× tryptone), indicating that it is not the lower nutrient content in tTBS *per se* that facilitates biofilm formation (see Fig. S1 in the supplemental material). In contrast, on yeast extract (YE)-only medium, the Δ*binK* mutant failed to form a biofilm in the absence of calcium, and in its presence, formed biofilms that lacked architecture (Fig. S1). Together, these data indicated that a nutrient(s) present in yeast extract is inhibitory to biofilm formation.

10.1128/mBio.02034-21.1 FIG S1 Yeast extract, but not tryptone, inhibits colony biofilms in the absence of calcium. Colony biofilm formation by the Δ*binK* mutant (KV7860) was evaluated following growth on 2× tryptone, 2× tryptone + 10 mM calcium, YE (LBS lacking tryptone), and YE + 10 mM calcium. Pictures were taken using a dissecting light microscope at 72 h before and after disruption using a toothpick. Pictures are representative of 3 separate experiments. Arrows indicate where “pulling,” indicating cohesion, was observed. Download FIG S1, TIF file, 2.9 MB. Copyright © 2021 Dial et al. 2021 Dial et al. https://creativecommons.org/licenses/by/4.0/ This content is distributed under the terms of the Creative Commons Attribution 4.0 International license.

Tryptone and yeast extract are largely undefined complex nutrient sources whose composition varies by batch and manufacturer, but one possible difference is the presence of specific vitamins in the latter (23). Investigating the potential impact of a variety of vitamins on biofilm formation resulted in the discovery that the addition of one, para-aminobenzoic acid (pABA), to tTBS was sufficient to inhibit biofilm formation by the Δ*binK* mutant; instead of forming cohesive colonies as it did on tTBS, the Δ*binK* mutant formed smooth, noncohesive colonies on tTBS that contained pABA (Fig. 1B). Furthermore, addition of calcium overrode the inhibition caused by pABA, permitting the characteristic Δ*binK* wrinkled colony to form, similar to those that form on LBS with calcium (compare Fig. 1B with Fig. 1A). Addition of pABA to liquid cultures was also insufficient to prevent calcium-induced biofilm formation (see Fig. S2 in the supplemental material). Thus, for the Δ*binK* mutant, calcium-mediated biofilm induction is dominant to pABA-based inhibition.

10.1128/mBio.02034-21.2 FIG S2 Biofilm induction by calcium is dominant to biofilm inhibition by pABA for the Δ*binK* mutant. The ability of pABA to inhibit biofilms formed by the Δ*binK* mutant (KV7860) during growth in liquid with shaking was assessed following growth in tTBS, tTBS + calcium, and tTBS + pABA/calcium. Pictures were taken after 16 h for cultures incubated at 24°C. Download FIG S2, TIF file, 2.8 MB. Copyright © 2021 Dial et al. 2021 Dial et al. https://creativecommons.org/licenses/by/4.0/ This content is distributed under the terms of the Creative Commons Attribution 4.0 International license.

### A combination of pABA and calcium promotes ES114 biofilm formation.

**FIG 2:**
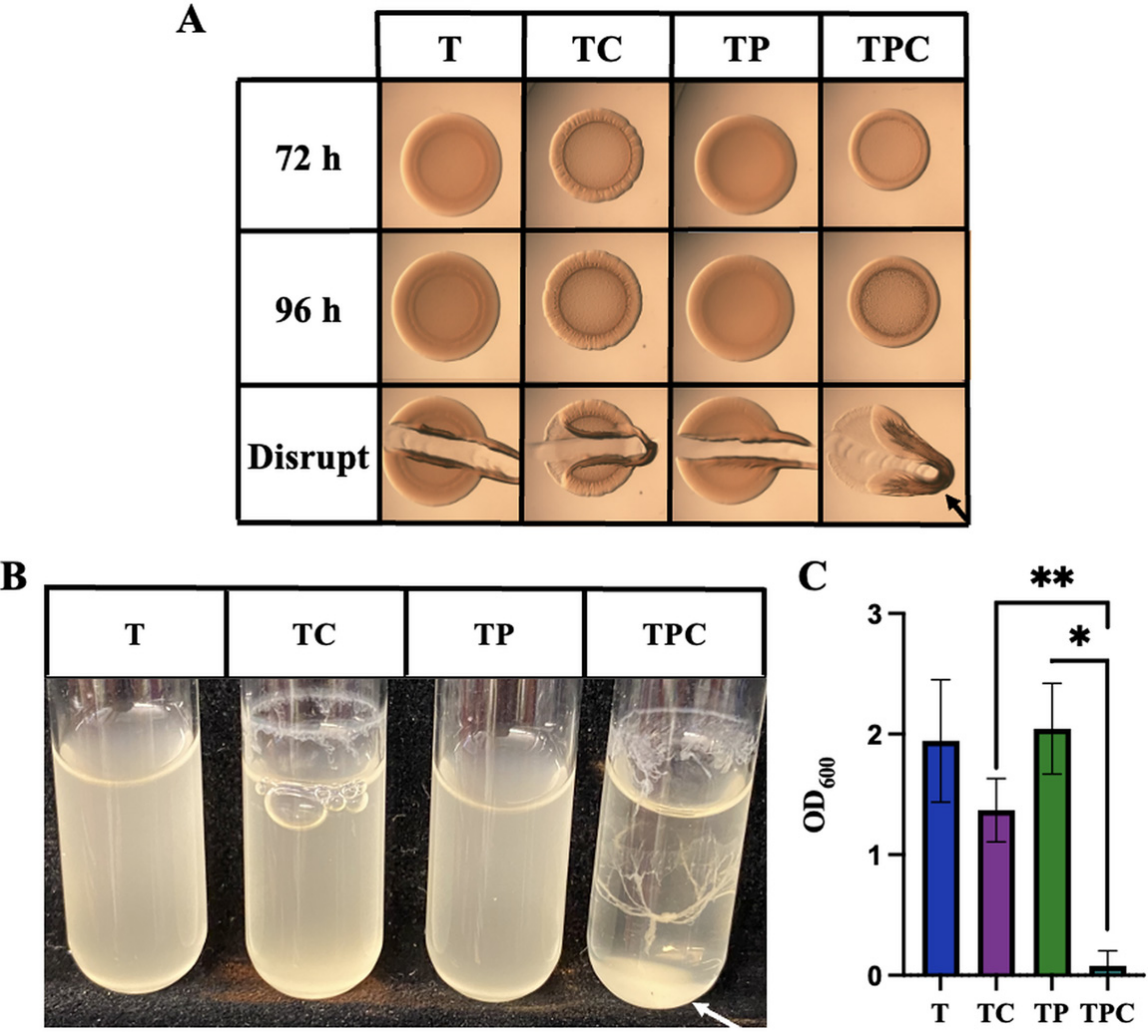
pABA induces ES114 biofilm. (A) Colony biofilm formation by wild-type strain ES114 was assessed following growth on tTBS (T), tTBS + 10 mM calcium (TC), tTBS + 9.7 mM pABA (TP), and tTBS + pABA/calcium (TPC). Pictures were taken using a dissecting light microscope at 72 and 96 h. The colony was disrupted using a toothpick after 96 h. (B and C) The ability of ES114 to produce biofilms during growth in liquid with shaking was evaluated following growth of the strain in tTBS, tTBS + calcium, tTBS + pABA, and tTBS + pABA/calcium. Pictures (B) and OD_600_ readings (C) were taken after 19 h. All pictures are representatives of 3 separate experiments. Arrows indicate where “pulling” as well as clumps, indicating cohesion, were observed. All experiments were done at 24°C. Statistics for C were performed via a one-way ANOVA using Tukey’s multiple-comparison test where OD_600_ was the dependent variable. **, *P* = 0.0098; *, *P* = 0.0360.

In the experiments described above, we included wild-type strain ES114 as a negative control, as this strain fails to form cohesive biofilms in the lab. Thus, we were surprised to find that, in the combined presence of pABA and calcium, ES114 produced cohesive colonies with modest architecture within 72 h (Fig. 2A). Furthermore, in liquid tTBS culture, the combined presence of pABA and calcium (pABA/calcium) resulted in “tree-like” structures similar to those that have previously been observed for the Δ*binK* mutant (Fig. 2B). We quantified the extent of biofilm formation by measuring the optical density at 600 nm (OD_600_) of the cultures, with the expectation that increased biofilm formation would result in decreased OD_600_. We observed that, while addition of calcium alone decreased the OD_600_, consistent with the readily observable biofilm ring above the liquid surface, the addition of pABA/calcium caused a further decrease in OD_600_, consistent with the observed ring and clump in that culture (Fig. 2B and D). Together, these data reveal, for the first time, conditions under which wild-type strain ES114 is competent to form a cohesive biofilm in laboratory culture.

To extend these findings, we asked whether pABA could induce ES114 biofilms in the complex medium LBS. However, pABA/calcium were unable to promote biofilm formation by LBS-grown ES114 (see Fig. S3 in the supplemental material). Thus, yeast extract appears inhibitory to ES114 biofilm formation; another vitamin or another as-yet unknown nutrient may also contribute to the biofilm-inhibitory nature of this medium.

10.1128/mBio.02034-21.3 FIG S3 ES114 fails to form colony biofilms on LBS supplemented with pABA/calcium. Colony biofilm formation by ES114 was evaluated following growth on tTBS + pABA/calcium (TPC) and LBS + pABA/calcium (LPC). Pictures were taken at 96 h before and after disruption with a toothpick. Arrows indicate where “pulling,” indicating cohesion, was observed. Download FIG S3, TIF file, 3.0 MB. Copyright © 2021 Dial et al. 2021 Dial et al. https://creativecommons.org/licenses/by/4.0/ This content is distributed under the terms of the Creative Commons Attribution 4.0 International license.

Together, these results indicate that pABA has the ability to both inhibit and induce biofilm formation, depending on the strain and growth condition. While this dual capability of pABA is intriguing, here, we chose to investigate the impact of pABA on the physiology of wild-type strain ES114. The ability of ES114 to form cohesive biofilms without genetic manipulation has not been previously reported and has the potential to greatly advance our understanding of the mechanisms of biofilm formation by this strain.

### Cellular aggregates form in the presence of pABA and calcium.

**FIG 3:**
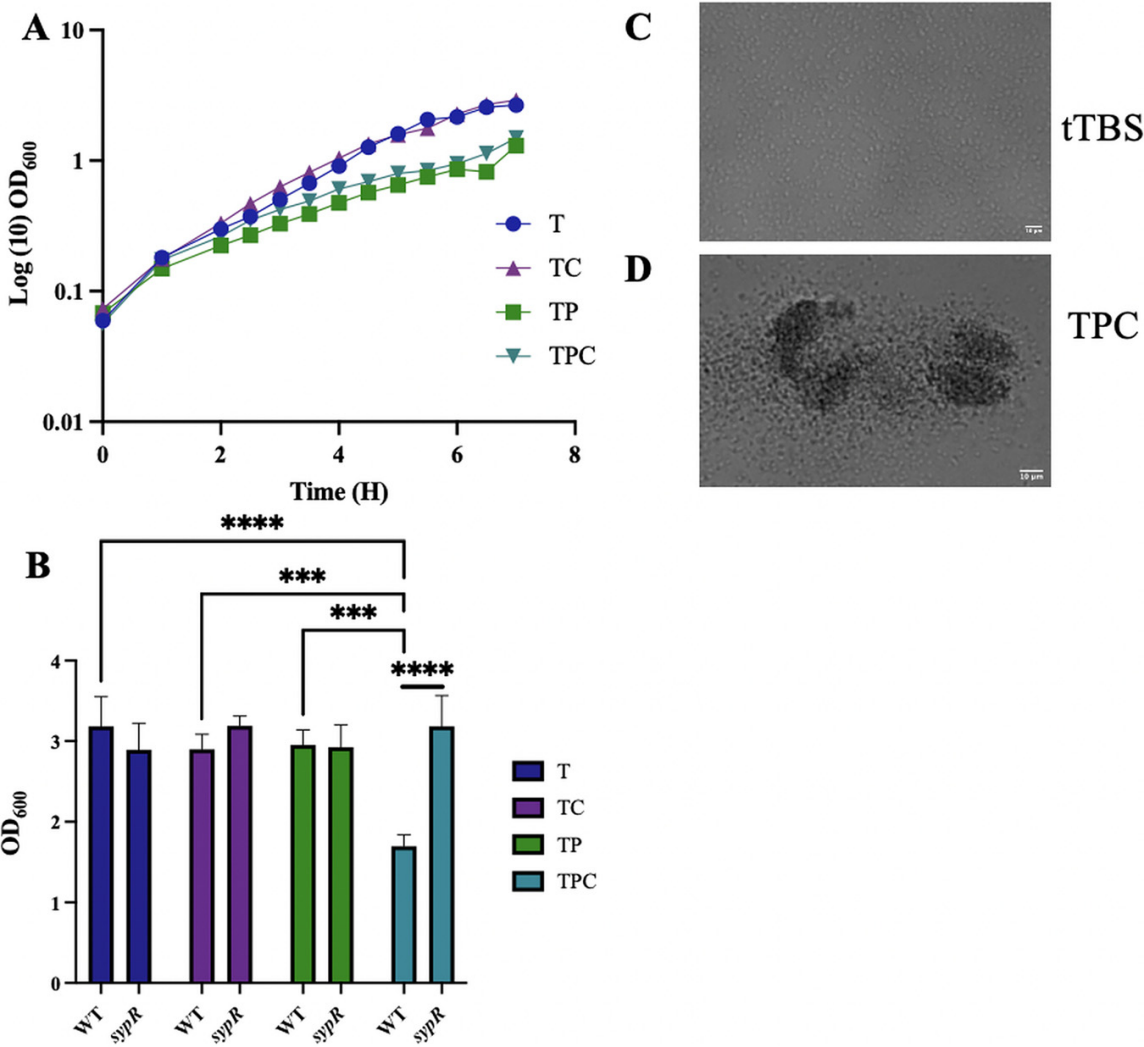
Cellular aggregates form in the presence of pABA and calcium. (A) Growth of WT ES114 was evaluated over time in tTBS (T), tTBS + calcium (TC), tTBS + pABA (TP), and tTBS + pABA/calcium (TPC) over the course of 7 h by monitoring the OD_600_ using a spectrophotometer. (B) Growth of WT ES114 and a Δ*sypR* mutant in T, TC, TP, and TPC after 19 h was monitored via OD_600_ using a spectrophotometer. Statistics for B were performed via a two-way ANOVA using Tukey’s multiple-comparison test for comparisons between media conditions (***, *P* < 0.0002; ****, *P* < 0.0001) and a 2-way ANOVA using Šídák’s multiple comparison test for comparisons between strains (****, *P* < 0.0001), where OD was the dependent variable in both comparisons. (C and D) Pictures of ES114 in T and TPC after 7 h of growth.

To understand the role of pABA, it was necessary to determine if the observed effects could be attributed to an impact of this vitamin on growth. Thus, we monitored growth of V. fischeri in tTBS alone or tTBS supplemented with calcium and/or pABA. Whereas calcium supplementation exerted no impact on growth, pABA addition (with or without calcium) modestly diminished the optical densities (OD) of the cultures, suggesting a slight growth defect (Fig. 3A). However, cells grown in the pABA condition achieved the same OD as those grown in tTBS within 19 h (Fig. 3B). While this was not the case for cells grown with pABA/calcium, the apparent growth defect in this condition could be attributed to the formation of aggregates, which were observed in the cultures containing pABA/calcium (Fig. 3C and D). To test whether cellular aggregation caused the decrease in OD, we evaluated growth using a mutant unable to produce *syp* biofilms, which as we demonstrate below are required for biofilm formation under these conditions; this strain achieved the same final OD in all growth conditions (Fig. 3B), indicating that biofilm formation accounted for the decreased OD observed for the wild-type strain at 19 h. Finally, the pH was not altered by pABA addition in these cultures but rather remained at the buffered pH of 7.5 (data not shown). These data suggest that the biofilm phenotypes can be attributed to a specific impact of pABA (and calcium) on biofilm formation rather than a growth defect.

### pABA- and calcium-induced biofilms rely on temperature.

**FIG 4:**
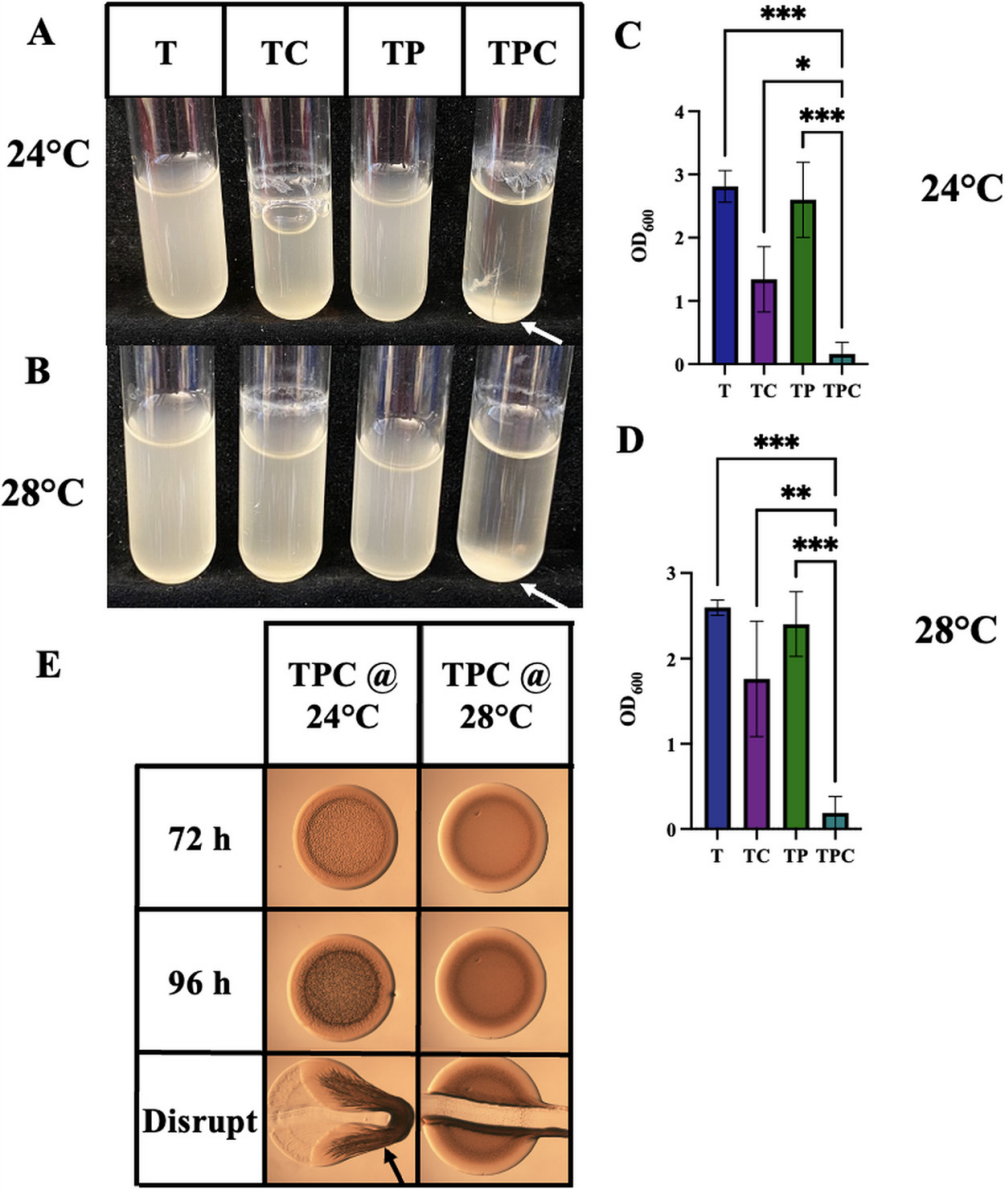
Impact of temperature on pABA/calcium-induced biofilms. (A to D) The ability of ES114 to form biofilms during growth in liquid with shaking biofilms was assessed following growth tTBS, tTBS + calcium, tTBS + pABA, and tTBS + pABA/calcium. Pictures (A and B) and OD_600_ readings (C and D) were taken after 16 h for cultures incubated at 24°C (A and C) or 28°C (B and D). All pictures are representatives of 3 separate experiments. Statistics for panels C and D were performed using one-way ANOVA using Tukey’s multiple-comparison test, where OD_600_ was the dependent variable. *, *P* < 0.05; **, *P* = 0.006; ***, *P* < 0.0007. (E) Colony biofilm formation by ES114 was assessed following growth on tTBS, tTBS + 10 mM calcium, tTBS + 9.7 mM pABA, and tTBS + pABA/calcium. Spotted colonies were incubated at both 24 and 28°C. Pictures were taken using a dissecting light microscope at 72 and 96 h. Each colony was disrupted using a toothpick after 96 h. Arrows indicate where “pulling” as well as clumps, indicating cohesion, were observed.

Most V. fischeri biofilm experiments, both those described above and in previous literature, have been performed at 24°C or room temperature, and some temperature-dependence has been noted, particularly in the case of *rscS* overexpression (6, 9, 11, 12). We, thus, wondered if temperature itself was a signal that would impact pABA- and calcium-induced biofilm formation by the wild-type strain. We found that, in shaking biofilm cultures, pABA/calcium could induce biofilm formation at 28°C to the same level as occurs at 24°C (Fig. 4A to D). In contrast, pABA/calcium were not able to induce biofilm formation on plates at 28°C (Fig. 4E). Thus, temperature seems to be a more significant factor controlling biofilm formation on solid media than in liquid, suggesting that additional biofilm control mechanisms exist on plates. All subsequent experiments were performed at 24°C.

### pABA- and calcium-induced ES114 biofilms require SYP.

**FIG 5:**
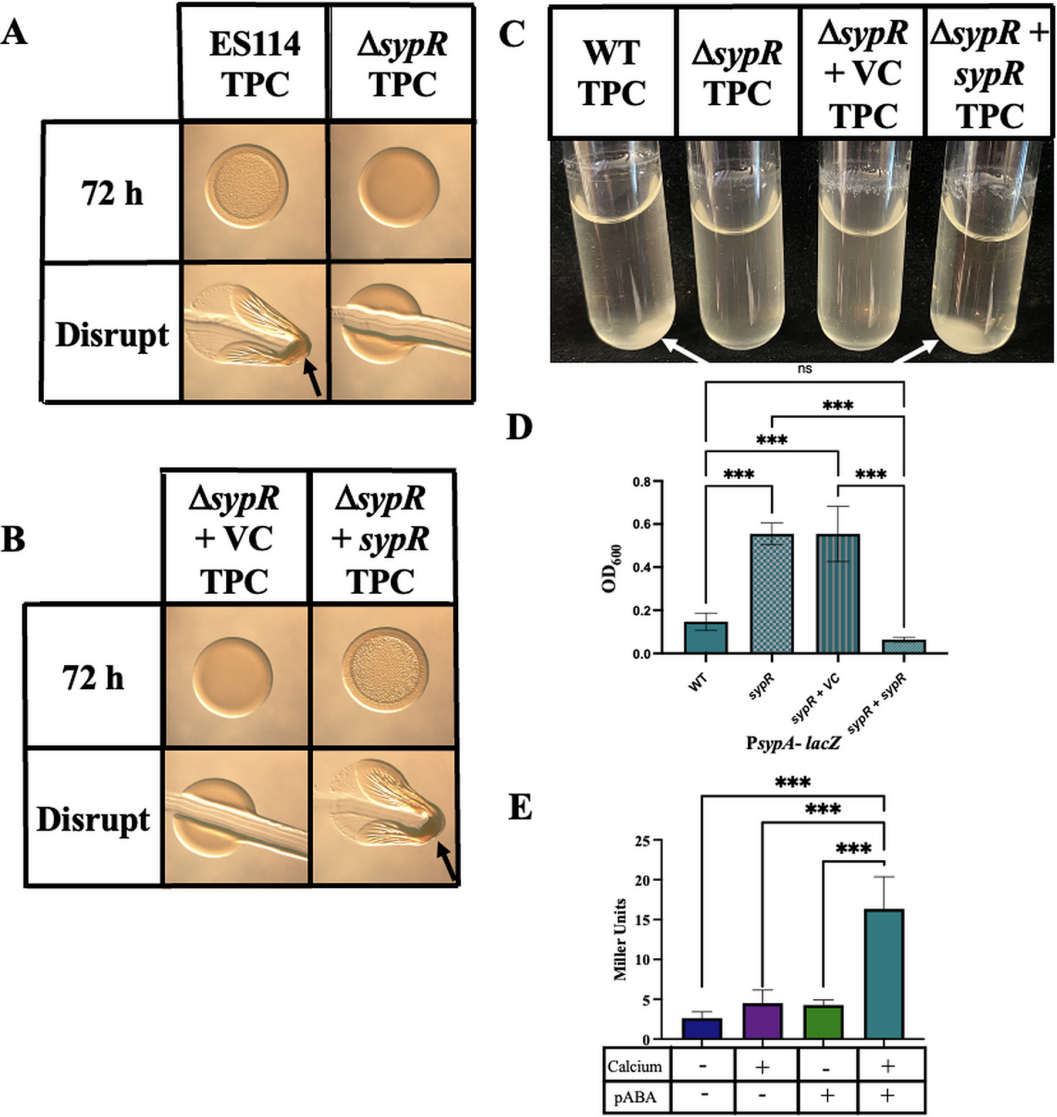
pABA- and calcium-induced ES114 biofilms require Syp. (A and B) Colony biofilm formation was assessed following growth of the indicated strains on tTBS + pABA/calcium (TPC). Pictures were taken at 72 h; at 72 h, each colony was disrupted using a toothpick and photographed. The following strains were tested: ES114, Δ*sypR* (KV5195), Δ*sypR* + vector control (VC), Δ*sypR* + *sypR*, and a plasmid-based *sypR* complement. Arrows indicate where “pulling” as well as clumps, indicating cohesion, were observed. (C and D) The same strains were evaluated for their ability for form biofilms in a shaking liquid culture. Pictures (C) were taken at 19 h, and OD_600_ of each culture (D) was measured at the same time as an indicator of biofilm formation. Statistics for D were performed via a one-way ANOVA using Tukey’s multiple-comparison test, where OD_600_ was the dependent variable. ***, *P* < 0.0006. (E) *sypA* promoter activity (Miller units) was measured using a P*sypA*-*lacZ* fusion strain (KV8079) following subculture for 22 h in tTBS, tTBS + calcium, tTBS + pABA, and tTBS + pABA/calcium. Statistics for panel E were performed via a one-way ANOVA using Tukey’s multiple-comparison test, where Miller units was the dependent variable. ***, *P* < 0.0006.

We next probed the roles of known polysaccharide loci in pABA-induced biofilms. Previous work has identified the *syp* locus as a key factor in the production by genetically altered strains of ES114 of robust, cohesive biofilms (5, 7). To determine if the pABA/calcium-induced ES114 biofilm formation depends on the *syp* locus, we evaluated an *sypR* deletion mutant, which is deficient in the ability to synthesize SYP. The Δ*sypR* mutant displayed a complete abrogation of cohesive biofilm formation on both solid media and in liquid cultures (Fig. 5A). Biofilm formation could be restored by complementation (Fig. 5A): the introduction of *sypR* on a plasmid, but not the corresponding vector control (VC) (pVSV105), was able to fully complement on plates and in liquid medium (Fig. 5B to D). These data, which use these new culture conditions to naturally induce biofilm in wild-type ES114, indicate that the observed biofilms are Syp dependent, providing support for the conclusions of previous studies that relied on genetically altered strains to produce cohesive colony biofilms that depend on Syp.

To begin to determine the underlying mechanism for pABA-induced biofilm formation, we asked if pABA and/or pABA/calcium could enhance *syp* promoter activity using a P*sypA*-*lacZ* fusion strain after 22 h. Basal levels of *sypA* promoter activity (5 Miller units) occurred in tTBS in the absence of supplementation (Fig. 5E). There was no substantial impact on *sypA* promoter activity with the addition of calcium or pABA alone. Upon the addition of both pABA and calcium, *sypA* promoter activity increased by about 3-fold to ∼15 Miller units. Though modest, this increase in *sypA* promoter activity by the addition of both nutrients may at least partially account for the increased biofilm formation observed under these conditions.

### Disruption of cellulose synthesis promotes cohesive biofilm formation by ES114.

**FIG 6:**
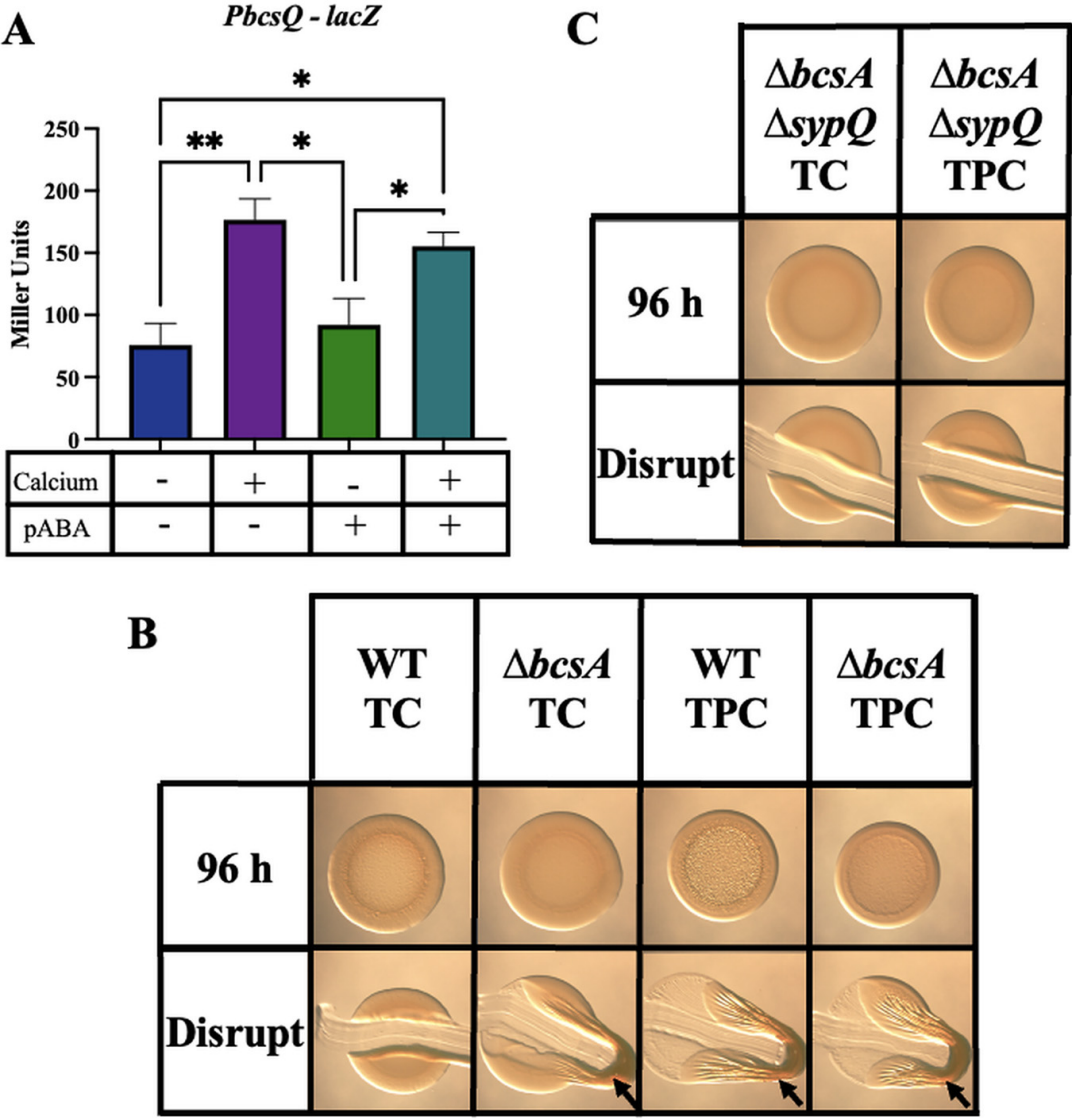
Disruption of cellulose synthesis promotes cohesive biofilm formation by ES114. (A) *bcs* promoter activity (Miller units) was measured using a P*bcsQ*-*lacZ* fusion strain (KV8078) following a 4-h subculture in tTBS, tTBS + calcium, tTBS + pABA, and T + pABA/calcium. Statistics for panel A were performed via a one-way ANOVA using Tukey’s multiple-comparison test, where Miller units was the dependent variable. *, *P* < 0.04; **, *P* = 0.0060. (B and C) Colony biofilm formation was assessed following growth of the ES114 and Δ*bcsA* (KV8616) strains (B) or the Δ*bcsA* Δ*sypQ* (KV9380) strain (C) on tTBS + calcium (TC) and tTBS + pABA/calcium (TPC). Pictures were taken using a dissecting light microscope at 96 h. Each colony was disrupted using a toothpick after 96 h. Arrows indicates where “pulling,” indicating cohesion, was observed.

Because cellulose (encoded by the *bcs* locus) also contributes substantially to biofilms that form during shaking liquid conditions, primarily by promoting ring formation (11, 24), we explored the impact of tTBS conditions on *bcs* promoter activity and the impact of cellulose on biofilms under these conditions. In tTBS-grown cultures, addition of calcium caused an increase in a *bcs* promoter reporter, similar to that previously reported in LBS conditions (Fig. 6A) (11). However, addition of pABA did not impact *bcs* promoter activity either alone or in the context of calcium, relative to the appropriate no-pABA controls. These data suggest that the impact of pABA on biofilm formation is not due to an effect on *bcs* promoter activity.

Despite the lack of impact of pABA on *bcs* promoter activity, we explored a possible role for *bcs* in ES114 biofilm formation using a Δ*bcsA* mutant, defective for cellulose production. Perhaps not unexpectedly, given the relative importance of SYP in cohesive biofilm formation, loss of BcsA did not diminish biofilm formation induced by pABA/calcium (Fig. 6B, right). However, unlike the wild-type strain, the *bcsA* mutant formed a colony biofilm on tTBS agar medium supplemented with calcium that was cohesive, albeit less so than that formed with pABA/calcium (Fig. 6B). This phenotype was not observed on LBS plates containing calcium (see Fig. S4 in the supplemental material). These results suggest that *bcsA*, or more likely cellulose production in general, inhibits *syp*-dependent biofilm formation under tTBS agar conditions. To determine if, in fact, the resulting coherent biofilms formed by the *bcs* mutant on tTBS supplemented with calcium were *syp* dependent, we evaluated a *bcs syp* double mutant. This double mutant failed to form a coherent biofilm (Fig. 6C). While the mechanism responsible for promoting biofilm formation by the *bcs* mutant under these conditions remains unknown, these data thus provide yet another condition, tTBS with calcium, that can provide insight into biofilm formation by specific strains of V. fischeri not observed when yeast extract is present.

10.1128/mBio.02034-21.4 FIG S4 A *bcsA* mutant fails to form cohesive biofilms on LBS or calcium-supplemented LBS. Colony biofilm formation by the Δ*bcsA* mutant (KV8616) was evaluated following growth on either LBS or LBS + calcium. Pictures were taken at 72 h and 96 h, following which the spots were disrupted with a toothpick. Download FIG S4, TIF file, 2.9 MB. Copyright © 2021 Dial et al. 2021 Dial et al. https://creativecommons.org/licenses/by/4.0/ This content is distributed under the terms of the Creative Commons Attribution 4.0 International license.

### pABA induces substantial transcriptional changes for biofilm and c-di-GMP-related genes.

To gain a global perspective on the impact of pABA on V. fischeri physiology, we performed a comparative transcriptome analysis with cells grown under the following conditions: tTBS, tTBS supplemented with calcium, and tTBS supplemented with both pABA and calcium. We isolated RNA from cultures at two key time points as follows: at 4 h, when calcium-induced cellulose production first becomes apparent as the formation of rings in the “splash zone” of shaking liquid cultures, and 8 h, when *syp*-dependent “trees” begin to form. Furthermore, for each 8-h biological replicate grown in tTBS with calcium/pABA, we separated the *syp*-dependent “tree” from the planktonic cells and sequenced each transcriptome independently.

**FIG 7:**
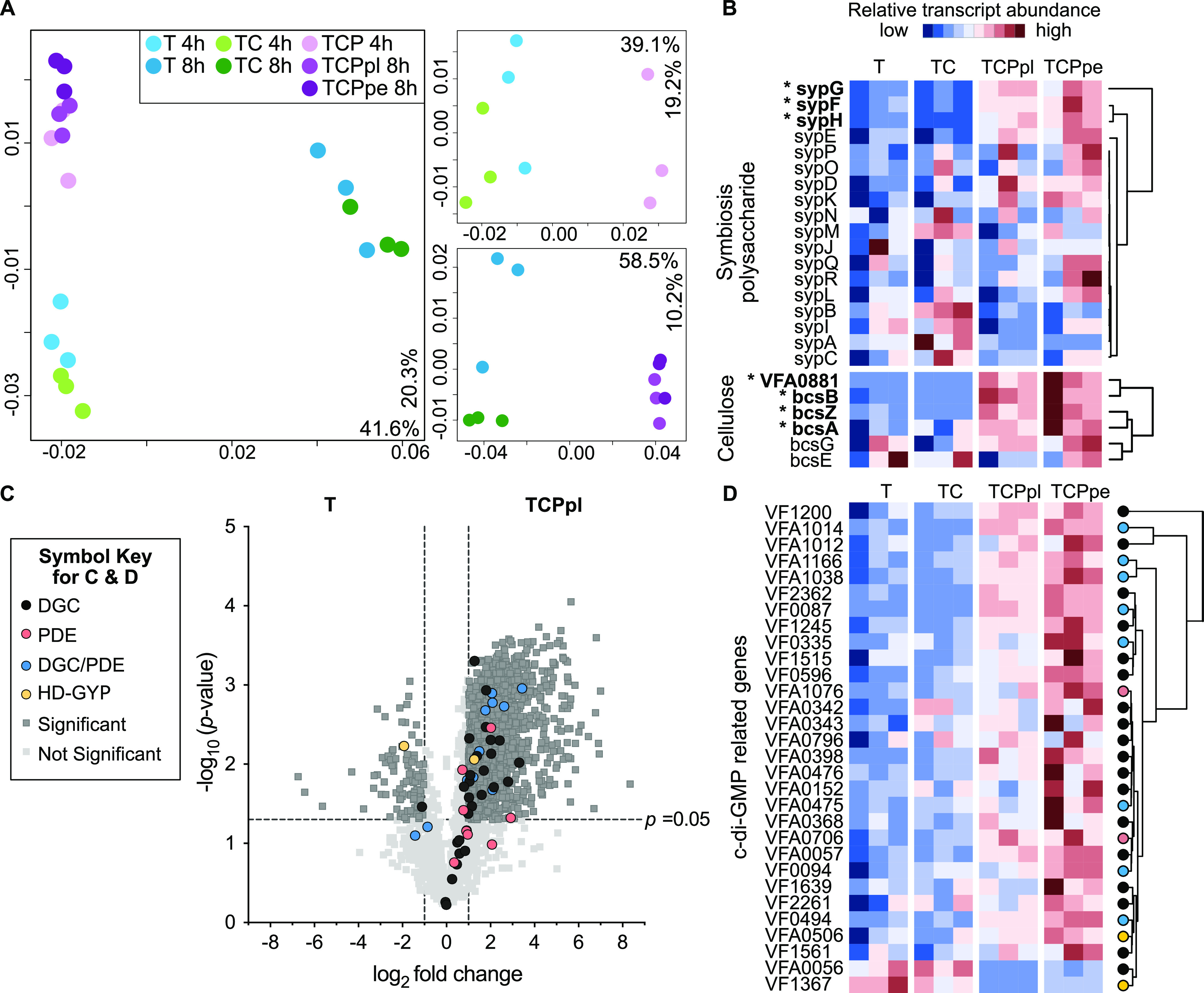
pABA and calcium promote expression of biofilm and c-di-GMP production transcripts. (A) Principal coordinate analysis (PCoA) plots based on Bray-Curtis dissimilarities of ES114 transcriptomes incubated in different media types at 4 and 8 h (left), 4 h only (right top), or 8 h only (right bottom). ES114 cells were incubated in tTBS (T) (blue), tTBS supplemented with 10 mM CaCl_2_ (TC) (green), or tTBS supplemented with 10 mM CaCl_2_ and 9.7 mM pABA (TCP) (purple). Cells in TCP were either collected from suspension and considered “planktonic” (TCPpl) or collected from the biofilm pellet at the bottom of the tube (TCPpe). Samples were collected after either 4 (lighter shade) or 8 h (darker shade) of incubation. Percentages on each axis indicate the amount of variation explained by each axis; *P* values indicate significant results of multivariate analysis of variance (PERMANOVA) tests. PCoA symbol key is shown above panel A. (B) Heatmap of hierarchical clustering results for the relative transcript abundance for ES114 8-h transcriptomes. Each row represents a gene from either the symbiosis polysaccharide (top) or cellulose biosynthesis (bottom) gene clusters; each column represents a sample. Square color in the heatmap indicates the relative transcript abundance for a given transcript across samples: red indicates high abundance, and blue indicates low abundance. Asterisks indicate genes that had significantly higher relative transcript abundance in TCPpl and TCPpe relative to T and TC treatments. (C) Volcano plot showing the log_2_ fold change in transcript abundance between ES114 cultures incubated in tTBS (T) or tTBS 10 mM calcium and 9.7 mM pABA planktonic cells (TCPpl) for 8 h. Transcripts with a negative log_2_ fold change value are more abundant in tTBS (left), and transcripts with a positive log_2_ fold change value are more abundant in tTBS calcium pABA (right). Symbols indicate the functional assignment of the gene/transcript of interest as follows: dark gray (DGC), red (PDE), yellow (HD-GYP), blue (DGC/PDE). Data points above the dashed horizontal line had significant *P* values between treatments (DESeq analysis; false-discovery rate [FDR], *P < *0.05), and those outside of the vertical dashed lines had a magnitude fold change of >|1| log_2_ between treatments (gray squares). (D) Heatmap of hierarchical clustering results for the relative transcript abundance for ES114 8-h transcriptomes. Each row represents a gene related to c-di-GMP production that was significantly differentially expressed in DESeq analysis (FDR, *P *< 0.05); each column represents a sample.

We began analyzing the transcriptome data using a principal coordinate analysis (PCoA) to determine how different the transcriptional profiles are among the seven treatments. PCoA performed on all treatments revealed that ES114 transcriptomes were influenced by both incubation time and the presence/absence of pABA (Fig. 7A). When samples were analyzed independently at each time point, samples clustered independently based on whether pABA was present in the media, with the presence/absence of pABA explaining 39.1% and 58.5% of the variation in the data set at 4 and 8 h, respectively (Fig. 7A).

We next focused on analyzing the relative transcriptional changes for biofilm-related genes across the four 8-h treatments. We chose to focus on the 8-h treatments because pABA accounted for a larger percentage of the variation in the data set at 8 h relative to 4 h. A hierarchical clustering analysis indicates cells grown with calcium and pABA are enriched in transcripts for most, but not all, *syp* and *bcs* genes, compared to the tTBS control (Fig. 7B). Genes whose transcripts were the most significantly enriched include *sypF*, *sypG*, *sypH*, *bcsA*, *bcsB*, and *bcsZ* and *VF_A0881*, which encodes a cellulose synthase protein. These results are consistent with the above findings that pABA and calcium contribute to enhanced transcription of the *syp* and *bcs* gene clusters (Fig. 5E and 6A).

We next turned to a more detailed comparison of transcriptomes from the 8-h time point for the tTBS control treatment and the calcium/pABA addition treatment to better understand how the cells respond to these biofilm-inducing conditions. The dual addition of calcium and pABA yielded over a thousand changes in gene expression, with the enrichment of transcripts for 688 genes and the depletion of transcripts for 659 genes in the calcium/pABA treatment relative to the tTBS control (Fig. 7C). When we mapped all 50 genes encoding proteins related to c-di-GMP synthesis and degradation onto our volcano plot showing all of the differentially expressed genes, 30 were significantly differentially expressed between the treatments (Fig. 7C). We next used a hierarchical clustering analysis to further explore the relative transcriptional changes for these 30 genes across all four treatments at the 8-h time point. Transcripts for 28 of the 30 genes were enriched in the calcium/pABA treatment (Fig. 7D), including 26 genes that encode putative DGCs or dual function enzymes that could contribute to the increased levels of c-di-GMP with pABA addition. Only two genes had transcripts that were significantly depleted in pABA/calcium, a predicted DGC and a predicted PDE (Fig. 7D). Finally, transcripts for the genes predicted to be involved in the uptake and subsequent metabolism of pABA, *VF_0639* and *pabC*, respectively, were also enriched in the calcium/pABA treatment relative to the tTBS control (see Table S1 in the supplemental material), suggesting that ES114 can uptake and metabolize pABA. Thus, pABA exerts a considerable impact on V. fischeri gene expression globally, most notably an enrichment of transcripts related to pABA transport and metabolism, cellulose and SYP biofilm, and c-di-GMP synthesis.

10.1128/mBio.02034-21.6 TABLE S1 Transcript differences of ES114 grown in tTBS with calcium/pABA relative to the tTBS control. Download Table S1, XLSX file, 0.7 MB. Copyright © 2021 Dial et al. 2021 Dial et al. https://creativecommons.org/licenses/by/4.0/ This content is distributed under the terms of the Creative Commons Attribution 4.0 International license.

### pABA upregulates c-di-GMP production.

**FIG 8:**
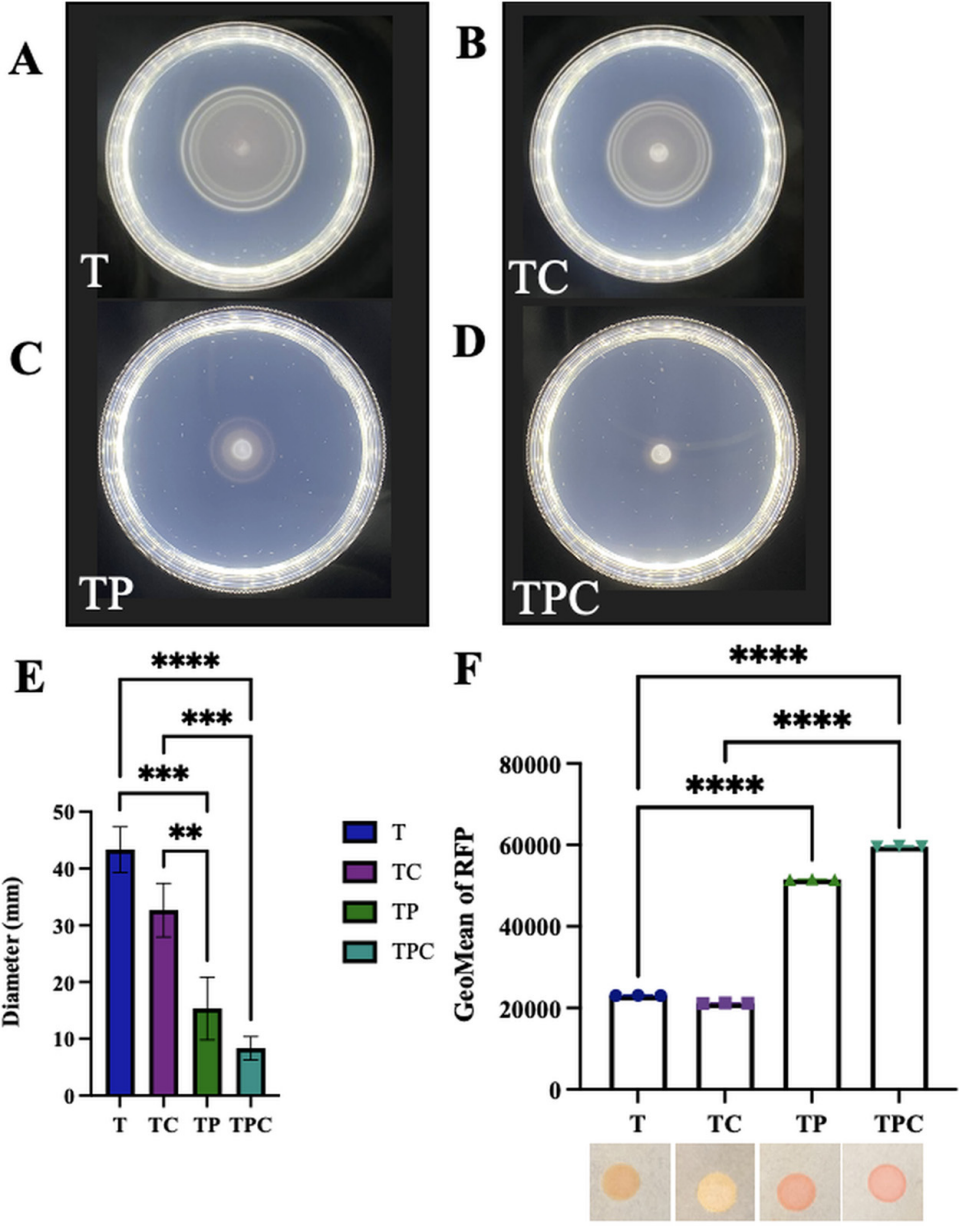
pABA controls motility and c-di-GMP production. (A to D) Migration of ES114 was evaluated using soft-agar motility plates supplemented with calcium, pABA, or both. Pictures were taken after 6 h, and representative images are shown. tTBS (T) (A), tTBS + calcium (TC) (B), tTBS + pABA (TP) (C), and tTBS + pABA/calcium (TPC) (D). (E) Migration was evaluated by measuring the outer diameter of the migrating cells. Statistics for panel E were performed via a one-way ANOVA using Tukey’s multiple-comparison test, where diameter was the dependent variable. **, *P* = 0.0049; ***, *P* < 0.0005; ****, *P* < 0.0001. (F) Levels of c-di-GMP were estimated using ES114 containing c-di-GMP biosensor plasmid pFY4535. RFP was measured from the same strain grown in T, TC, TP, or TPC liquid cultures using flow cytometry. The cells were first gated using AmCyan and then on RFP. Statistics for panel F were performed via a one-way ANOVA using Tukey’s multiple-comparison test. ****, *P* < 0.0001. A culture of this strain was spotted onto tTBS plates containing calcium, pABA, or pABA/calcium, and the resulting RFP production was visualized as spots with different shades of pink that mirrored the flow cytometry measurements. Representative pictures are shown.

Because pABA/calcium addition resulted in enriched transcripts for many c-di-GMP-related genes, we hypothesized that c-di-GMP could play a role in the pABA- and calcium-induced phenotypes. c-di-GMP is a second messenger that promotes biofilm formation and inhibits motility. Because of this dichotomy, we asked if pABA supplementation impacted motility of ES114. ES114 migrates rapidly in a semisolid medium, reaching a diameter of around 45 mm within 6 h (Fig. 8A, B, and E). In contrast, when pABA was added to the medium, the migration was drastically slowed, and ES114 only reached a diameter of 8 mm within the same 6-h time frame (Fig. 8C to E). These results provided support for the potential involvement of c-di-GMP.

To determine if pABA addition increases c-di-GMP levels, we used a c-di-GMP biosensor that contains the *rfp* gene (encoding red fluorescent protein) under the control of a c-di-GMP-binding riboswitch (25). Increased levels of c-di-GMP result in increased production of RFP, which can be visualized and quantified. Addition of pABA to cultures of ES114 containing the biosensor produced a visibly and measurably significant increase in RFP production relative to unsupplemented cultures (Fig. 8F), indicating an upregulation of c-di-GMP production. These data are consistent with the differential impact of pABA on biofilm formation and motility. While c-di-GMP levels were not substantially impacted by the addition of calcium alone, there was a trend toward increased levels that did not reach the levels of significance in the presence of both pABA and calcium. Notably, when biofilm formation by the reporter strain was assayed in the presence of calcium and pABA, the strain formed clumps that were a bright pink, indicating high-levels of c-di-GMP being produced within the cells of the biofilm (see Fig. S5 in the supplemental material).

10.1128/mBio.02034-21.5 FIG S5 Biofilm “clumps” produce c-di-GMP. ES114 containing c-di-GMP biosensor plasmid pFY4535 produces visually pink clumps from production of RFP following overnight growth in tTBS + pABA/calcium. Arrows indicate where clumps, indicating cohesion, were observed. Download FIG S5, TIF file, 2.8 MB. Copyright © 2021 Dial et al. 2021 Dial et al. https://creativecommons.org/licenses/by/4.0/ This content is distributed under the terms of the Creative Commons Attribution 4.0 International license.

### c-di-GMP promotes *syp*-dependent biofilm phenotypes.

**FIG 9:**
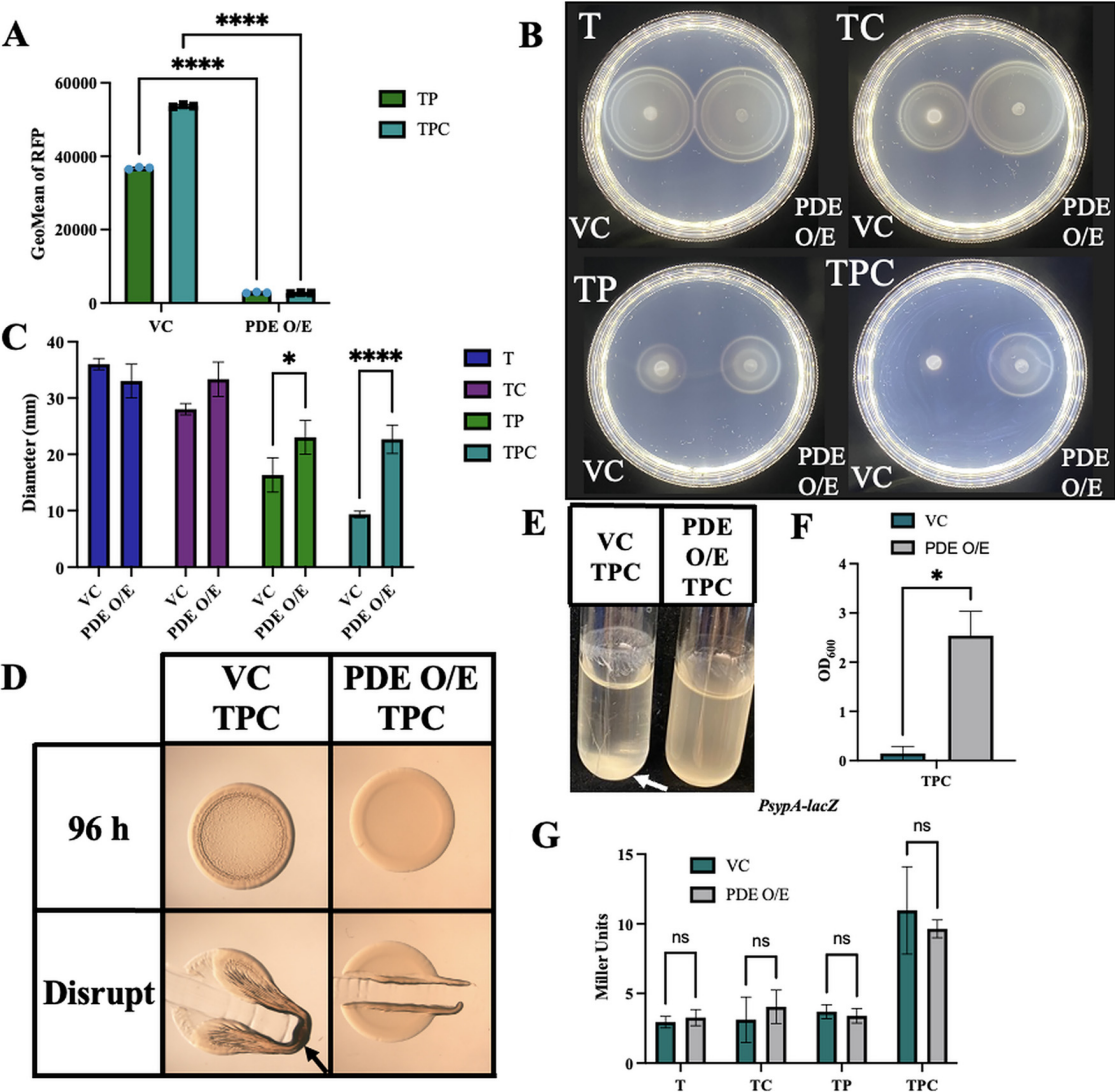
PDE overexpression abrogates c-di-GMP levels and biofilm formation but has less effect on motility and no effect on *syp* transcription. (A) The levels of RFP from strains carrying c-di-GMP biosensor pFY4535 and either the vector control or a plasmid (pKV302) that overexpresses phosphodiesterase (PDE) VF_0087 were measured using flow cytometry following growth in either tTBS + pABA (TP) or tTBS + pABA/calcium (TPC). The cells were first gated on AmCyan and then on RFP. Statistics for panel A were performed via a one-way ANOVA using Tukey’s multiple-comparison test. ****, *P* < 0.0001. (B and C) Migration of ES114 carrying vector control (VC) or PDE overexpressing plasmid pKV302 (PDE O/E) was evaluated using tTBS soft-agar motility plates (T) supplemented with calcium (TC), pABA (TP), or both (TPC). Pictures were taken after 6 h, and representative images are shown. Migration was evaluated by measuring the outer diameter of the migrating cells. Statistics for panel C were done via a 2-way ANOVA using Šídák’s multiple comparison test, where diameter was the dependent variable. *, *P* = 0.0137; ****, *P* < 0.0001. (D) Colony biofilm formation was assessed following growth of ES114 carrying the vector control (VC) or pKV302 (PDE O/E) on tTBS + pABA/calcium (TPC). Pictures were taken at 96 h before and after disrupting the spots with a toothpick. (E and F) The same strains in panel D were grown in tTBS liquid medium containing pABA/calcium with shaking. Pictures were taken at 19 h, and the OD_600_ was measured as an indicator of biofilm formation. Arrows indicate where “pulling” as well as clumps, indicating cohesion, were observed. Statistics for panel F were performed using a paired *t* test, where OD_600_ was the dependent variable. *, *P* = 0.0136. (G) *sypA* promoter activity (Miller units) was measured using P*sypA*-*lacZ* fusion strain KV8079 that contained either the VC plasmid or the PDE O/E plasmid following a 22-h subculture in T, TC, TP, and TPC. Statistics for panel G were performed via a two-way ANOVA using Šídák’s multiple comparison test, where Miller units was the dependent variable.

Because a role for c-di-GMP in *syp*-dependent biofilm formation has not been previously reported, we sought to determine if the ability of pABA to induce biofilm formation is reliant on c-di-GMP. To test this possibility, we overexpressed the gene for a c-di-GMP phosphodiesterase (PDE), VF_0087, which we have previously shown to be effective in decreasing c-di-GMP levels of strain KB2B1 (26). First, we asked if overexpression of *VF_0087* abolished or diminished the pABA-induced increase in c-di-GMP using the biosensor. Indeed, we found that overexpression of *VF_0087* significantly decreased the levels of RFP, indicating decreased levels of c-di-GMP (Fig. 9A). Second, we asked if overexpression of *VF_0087* increased migration of ES114 through soft agar supplemented with pABA and found that it partially rescued ES114’s pABA-induced migration defect (Fig. 9B and C). It also rescued ES114’s calcium-induced migration defect. Because the overexpression of *VF_0087* substantially decreased c-di-GMP levels without fully restoring motility, we conclude that the impact of pABA on migration may be multifactorial. Third, we asked if overexpression of *VF_0087* could inhibit biofilm formation induced by pABA/calcium. Whereas the vector control strain produced cohesive colony biofilms, the PDE-overproducing strain failed to form a biofilm on tTBS plates supplemented with pABA/calcium (Fig. 9D). Fourth, we looked at whether PDE overexpression would also inhibit biofilm formation in shaking cultures. We observed a trend similar to that on plates, with the vector control strain producing clumps, rings, and trees and the *VF_0087*-overexpressing strain lacking these biofilms (Fig. 9E and F). Finally, we asked if *VF_0087* overexpression affected *syp*-dependent biofilms by controlling *sypA* promoter activity. Although we found that PDE overexpression did not have any impact on *sypA* promoter activity at 22 h (Fig. 9G), it is possible that c-di-GMP exerts its effect on SYP biofilm by mediating transcriptional changes of other key *syp* genes and/or at the posttranscriptional level. These data reveal that pABA-induced c-di-GMP is a key underlying mechanism controlling the ability of ES114 to form *syp*-dependent biofilms.

## DISCUSSION

Past studies of biofilm formation by V. fischeri have depended on genetically engineered strains, such as an *rscS*-overexpressing strain or a Δ*binK* mutant, due to their ability to produce substantial biofilm formation in laboratory culture, unlike wild-type strain ES114. This will no longer be necessary, as our findings here revealed conditions under which wild-type ES114 is competent to form cohesive, *syp*-dependent biofilms in the laboratory within as few as 8 h of culturing without genetic manipulation, although substantial induction of some genes such as *sypA* may take longer, i.e., 22 h. These conditions induced numerous changes in gene expression, including the enrichment of transcripts for over 100 transcriptional regulators, and resulted in a substantial increase in c-di-GMP levels that proved necessary for *syp*-dependent biofilm formation. We anticipate that these conditions will facilitate further investigation of signals and processes critical to biofilm formation by V. fischeri.

Although pABA seems like an unusual signal molecule, its role in inducing biofilm formation has been documented previously in the multispecies biofilms formed by oral pathogens Streptococcus gordonii and Porphyromonas gingivalis (27). S. gordonii produces and secretes pABA into the environment. In turn, P. gingivalis metabolizes the exogenous pABA, leading to increased expression and production of the fimbrial adhesins necessary for colonization and, thus, increased ability to adhere in the mixed-species biofilm. Furthermore, pABA treatment resulted in increased colonization of P. gingivalis in a mouse model of infection (27). Proteomic studies revealed that pABA exposure resulted in changes in the levels of hundreds of proteins, indicating a substantial response, similar to what we observed via transcriptome analysis for V. fischeri. This study thus provides evidence that pABA can be an environmental signal that induces key changes in bacterial behavior, namely, the induction of biofilm behaviors in P. gingivalis, as we saw also in V. fischeri. Whether pABA is produced and used as a signal in the context of the squid host of V. fischeri remains to be determined.

Like S. gordonii, Escherichia coli secretes pABA; these cells can also take up this small molecule. In E. coli, the AbgT family of transporters is responsible for the uptake of pABA in its glutamate-bound form (pABA-glu) and subsequent cleavage of the glu residue. In addition, free pABA can enter cells through both transport by AbgT and membrane diffusion (28). In our transcriptome experiment, pABA/calcium conditions resulted in the enrichment of an AbgT family transporter gene *VF_0639*. However, our preliminary data revealed no biofilm defect for a mutant deleted for that putative *abgT* transporter gene (C. N. Dial and K. L. Visick, unpublished data). Therefore, while it seems likely that V. fischeri can uptake pABA, resulting in robust biofilm formation, we anticipate that the transport may occur via multiple routes similar to E. coli.

Whereas pABA alone is sufficient to induce biofilm formation by S. gordonii, in V. fischeri strain ES114, neither pABA nor calcium (in tTBS) alone was sufficient to induce *syp* transcription or *syp*-dependent biofilm formation; coordinate induction by both signals was necessary. Coordinate signaling is a common theme in bacteria. For example, in Pseudomonas aeruginosa, sensor kinases LadS and GacS, recognize signals and relay them through GacA to eventually promote biofilm formation and chronic infection (29, 30). Coordinate or synergistic signaling is also a well-established method for luminescence control in *Vibrio* species, including Vibrio harveyi and V. fischeri, where two autoinducers can signal through separate two-component sensors to induce bacterial luminescence (31, 32).

Calcium is abundant in seawater at concentrations similar to those used here (10 mM), making it a physiologically relevant signaling molecule for V. fischeri in its natural environments. The specific role of calcium in biofilm formation was not readily apparent from our transcriptome experiment; however, we anticipate that it may function to control multiple pathways. There are increasing examples of the importance of calcium signaling in bacteria in a variety of pathways via regulation at both transcriptional and posttranscriptional levels. For example, in P. aeruginosa and Pseudomonas syringae, calcium activates transcription of genes for surface adhesins and exopolysaccharide (EPS) production (33, 34). Calcium directly interacts with proteins, such as the pilus-biogenesis factor PilY1 in P. aeruginosa, which enables pilus protraction and retraction; its interactions with type I pili promote E. coli to initiate entry into host cells (35, 36). In V. vulnificus, calcium (10 mM) increases the levels of c-di-GMP, which then triggers production of the *brp* polysaccharide by upregulating transcription of the *brp* locus (37). The same amount of calcium failed to exert a big effect on c-di-GMP levels (or *syp* transcription) under the conditions assayed here. In contrast, pABA caused a substantial increase in c-di-GMP levels regardless of the presence of calcium. Because calcium was also necessary for biofilm induction despite the pABA-mediated induction of c-di-GMP, calcium must play a distinct and necessary role in inducing biofilm formation in V. fischeri, potentially at a posttranscriptional level.

Likewise, the specific role of pABA in promoting biofilm formation is somewhat unclear. pABA supplementation increased the transcript levels of numerous *syp* genes as well as DGCs that correspondingly caused an increase in levels of c-di-GMP that was required for biofilm formation. There are a number of examples in which increased c-di-GMP leads to increased transcription of genes for exopolysaccharide synthesis, such as in P. aeruginosa and Vibrio cholerae. In P. aeruginosa, c-di-GMP binds the transcriptional regulator FleQ and promotes the transcription of the *pel* and *psl* genes, resulting in the upregulation of the exopolysaccharides PEL and PSE and subsequent biofilm formation (38–40). Similarly, in V. cholerae c-di-GMP regulates biofilm formation transcriptionally via VpsR and VpsT; c-di-GMP directly binds to these regulators to activate the expression of the *vps* genes and upregulate biofilm formation (41). However, increased c-di-GMP did not appear to be responsible for inducing *sypA* transcription, as PDE overexpression failed to substantially diminish *sypA* promoter activity; thus, it is likely pABA contributes to induction of biofilm formation in an additional way(s) and/or that our assay conditions do not allow us to accurately measure control over *syp* transcription exerted by c-di-GMP. c-di-GMP may promote SYP biofilms posttranscriptionally, as seen in other bacterial species. For example, c-di-GMP binds to and activates the cellulose synthase complex of BcsA-BcsB to produce and translocate cellulose in a number of bacteria, including E. coli, Komagataeibacter xylinus, and Rhodobacter sphaeroides (42, 43). c-di-GMP controls the production of curli in both E. coli and Salmonella enterica via the upregulation of *csgD* both transcriptionally and posttranscriptionally. CsgD, in turn, upregulates the *csgBAC* operon, which encodes the structural subunits of curli, thus increasing biofilm formation (44, 45). Future work will be required to determine how pABA signaling through c-di-GMP-dependent and -independent mechanisms coalesce to produce SYP biofilm.

Our work also revealed a complex interaction between the two major polysaccharides, Syp and cellulose. In shaking liquid cultures, the two polysaccharides contribute to biofilm formation, with Syp associated with cohesive cellular clumps at the bottom of the test tube, cellulose responsible for adherence in a ring around the perimeter, and both contributing to “tree” formation (11) (Fig. 3). In contrast, deletion of the cellulose synthesis gene *bcsA* permitted Syp-dependent colony biofilms to form with calcium supplementation alone (rather than requiring pABA and calcium). These data suggest that, under these conditions, cellulose production could inhibit Syp production or export. This phenomenon of one type of polysaccharide inhibiting the production of the others also occurs in P. aeruginosa: loss of production of the exopolysaccharide PSL results in overproduction of the polysaccharide PEL (46). We anticipate that the use of calcium-supplemented tTBS conditions will uncover additional factors that contribute to control over production of the two polysaccharides.

In summary, our work identified two signals, pABA and calcium, that coordinately induce biofilm formation by ES114 *in vivo*. These findings permitted us to uncover a link between the signaling molecule c-di-GMP and Syp-dependent biofilm formation. This work also revealed a complex interaction between the two major polysaccharides involved in biofilm formation, Syp and cellulose. The conditions established here thus represent a significant advance that will allow us and others to investigate control over biofilm formation without relying on genetic manipulation, which will ultimately facilitate discovery of additional natural signals that control this important trait.

## MATERIALS AND METHODS

### Strains and media.

V. fischeri strains used in this study are shown in Table 1. Plasmids used in the study are shown in Table 2. Wild-type ES114 was the parent strain used in this study. E. coli strains were grown in lysogeny broth (LB) (1% tryptone, 0.5% yeast extract, and 1% sodium chloride) (10). V. fischeri strains were cultured in either Luria-Bertani salt (LBS) medium (1% tryptone, 0.5% yeast extract, 2% sodium chloride, and 50 mM Tris, pH 7.5) or Tris-buffered TBS (tTBS) (1% tryptone, 2% sodium chloride, and 50 mM Tris, pH 7.5) where noted. All assays on solid media were performed using Gibco (Difco) tryptone, and all assays in liquid media were performed using Fisher tryptone. To test the possible role of vitamins in inhibiting biofilm formation, tTBS medium was supplemented individually with the following vitamins at various concentrations: nicotinic acid, choline, inositol, para-aminobenzoic acid, pyridoxine, folic acid, and thiamine. The concentration of pABA used here (9.7 mM) was determined empirically as the amount sufficient to promote biofilm formation while not substantially impairing bacterial growth. For motility experiments, cells were grown in Tris-buffered saline (TBS) broth (1% tryptone, 2% sodium chloride, and buffered with 50 mM Tris, pH 7.5, when pABA was added) (10) and inoculated onto TBS soft agar plates that were solidified with 0.25% agar and supplemented with 35 mM MgSO_4_ and either 10 mM CaCl_2_ and/or 9.7 mM pABA where noted (10). For growth in minimal medium, Tris-minimal medium (TMM) was used (10). For V. fischeri, antibiotics were used as follows: tetracycline (Tet), 2.5 μg/ml; kanamycin (Kan), 100 μg/ml; chloramphenicol (Cm), 5 μg/ml; gentamycin (Gent) 10 μg/ml. For E. coli, antibiotics were used as follows: Cm, 12.5 μg/ml; Kan, 50 μg/ml; and Gent 10 μg/ml. For growth of E. coli thymidine auxotroph strain π3813 (47), which carries the conjugal plasmid pEVS104 that was used to facilitate conjugations, thymidine (Thy) was added to a final concentration of 0.3 mM.

**TABLE 1:** Strains used in this study

Strain	Genotype	Reference
ES114	Wild type	57
KV5195	Δ*sypR*	7
KV7860	Δ*binK*	11
KV8078	Δ*sypQ*::Cm *att*Tn*7*:: *pbcsQ-lacZ*	11
KV8079	Δ*sypQ*::FRT-Cm IG (yeiR-glmS)::*PsypA lacZ att*Tn7::Erm	11
KV8616	Δ*bcsA*::FRT-Trim	58
KV9380	Δ*bcsA*::FRT Δ*sypQ*::FRT	58

**TABLE 2:** Plasmids used in this study

Plasmid	Description	Reference
pEVS104	Conjugal helper plasmid (Kan^r^)	59
pFY4535	c-di-GMP biosensor plasmid that encodes RFP under the control of a c-di-GMP-dependent riboswitch and AmCyan under the control of a constitutive promoter	25
pKV69	Vector control	60
pKV302	pKV69 + *VF_0087*	26
pSS22	pVSV105 containing *sypR*	7
pVSV105	Stable expression vector (Cm^r^)	61

### Plasmid conjugation.

Bacterial conjugation was used to transfer the plasmids of interest into the strains noted as described previously (48). Briefly, the recipient V. fischeri strains were inoculated into 5 ml of LBS and grown overnight at 28°C. Donor E. coli strains (carrying the plasmid of interest) were inoculated into LB with the appropriate supplements (antibiotics), and the helper E. coli strain was inoculated into LB with the appropriate supplements (Thy and Kan) and grown overnight at 37°C. The strains were then subcultured, in the same growth conditions, and grown to early exponential phase. Then, 1 ml of the V. fischeri recipient and 250 μl of each of the E. coli strains were added to 1.5-ml microcentrifuge tubes and concentrated using a tabletop microcentrifuge for 2 min at 13,300 rpm at room temperature (48). Separately, as a negative control, each V. fischeri recipient culture was also concentrated alone by centrifugation as described above. Supernatants were decanted, and the remaining liquid was used to resuspend the pellets at the bottom of the tube. An aliquot (∼15 μl) was then spotted onto LBS without antibiotics and placed into the 28°C incubator for a minimum of 3 h or overnight. The colonies were then streaked onto media with the appropriate antibiotics and grown overnight at 28°C. The resultant colonies were then restreaked onto the appropriate media, and the individual colonies were grown in liquid cultures and saved.

### Wrinkled colony assays.

One day prior to experimentation, either LBS (+/− Ca) or tTBS plates (+ Ca, + pABA, and + Ca + pABA) were made by pipetting 25 ml of the appropriate medium into petri dishes and left to dry overnight. Cultures were inoculated from frozen and grown overnight in 5 ml of tTBS with shaking at 28°C. The strains were then subcultured (100 μl into 5 ml tTBS) and grown with shaking at 28°C for 1 to 2 h. Next, the cultures were normalized to an OD_600_ of 0.2 in tTBS. The normalized cultures were then spotted onto the tTBS plates with or without additives at 10 μl per spot and left to dry completely before inverting and incubating at 24°C or 28°C where noted. After 24, 48, 72, and/or 96 h, the spots were assessed under a dissecting microscope and photographed before and after being disrupted with a toothpick; this “toothpick” assay permits an assessment of the relative stickiness of the colony biofilm (49).

### Liquid/shaking biofilm assay.

The denoted strain was inoculated in 5 ml of either LBS or tTBS from a frozen stock and grown with shaking at 28°C overnight (∼16 h). The next day, 2 ml of the appropriate medium (e.g., tTBS, tTBS + 10 mM calcium, tTBS + 9.7 mM pABA, and tTBS + calcium and pABA) were inoculated with 2 μl of the overnight culture. The tubes were then incubated with shaking at 24°C for 14 to 20 h. After the designated incubation period, the tubes were gently removed from the shaker (so as not to disrupt biofilm formation) and pictures were taken using an iPhone camera. For quantifying the biofilm, a volume of 100 μl to 1 ml of the culture was added to a cuvette and tTBS was added as needed for a final volume of 1 ml. The OD_600_ was measured using a spectrophotometer in triplicate, and the OD_600_ was adjusted for the appropriate dilution factor (1:10).

### Growth curves.

Cells were grown overnight at 24°C in tTBS medium. In the morning, the strains were subcultured to a starting OD_600_ of 0.05 in tTBS and grown with shaking at 24°C in 250 ml baffled flasks. Aliquots of cultures (1 ml) were taken at 30-min to 1-h intervals, and the OD_600_ was measured using a spectrophotometer. Cultures were diluted for more accurate measurements when the OD_600_ was above 1.

### β-Galactosidase assays.

Reporter strains were streaked onto tTBS plates and grown at 28°C overnight (∼16 h). A single colony was then picked and used to inoculate 5 ml tTBS in 18- by 150-mm tubes. Three different colonies were used for three different replicates, and these tubes were grown at 24°C with shaking overnight. In the morning, all three replicates of the reporter strain were subcultured in 125-ml baffled flasks with 20 ml of the 4 different media types (tTBS, tTBS + calcium, tTBS + pABA, and tTBS + calcium + pABA). For the P*sypA-lacZ* reporter, strains were grown for 22 h, while the P*bcs-lacZ* reporter strains were subcultured for 3 h. The final concentrations of calcium and pABA were 10 mM and 9.7 mM, respectively. After the indicated growth period, an aliquot (5 ml) of each culture was concentrated by centrifugation, and a β-galactosidase assay (Miller assay [50]) was performed as previously described (11). The OD_420_ and OD_550_ were then measured using a 96-well plate reader, and Miller units were calculated as previously described (11).

### Motility assays.

Bacteria were grown overnight at 28°C in TBS and then subcultured in the same medium and allowed to grow to an OD_600_ of between 0.2 and 0.4. The cultures were then normalized to an OD of 0.2, and 10-μl aliquots were spotted onto soft-agar motility plates (TBS-Mg) with or without 10 mM calcium and/or 9.7 mM pABA. The plates were then incubated at 28°C for 6 to 8 h. Spot diameter measurements were taken at the times indicated, and pictures were taken with plates illuminated from below using an iPhone camera.

### Visualization of c-di-GMP-induced RFP production.

From frozen stock, ES114 containing pFY4535 (25) was inoculated into 5 ml of tTBS and grown overnight in the 28°C incubator. pFY4535 contains genes for AmCyan, which is under the control of a constitutive promoter, and RFP, which is under the control of a c-di-GMP-dependent riboswitch. The next day, the cultures were subcultured for 2 h, normalized to an OD_600_ of 0.2, and spotted on tTBS medium containing either 10 mM calcium, 9.7 mM pABA, or both. The plates were then placed in the 24°C incubator for 24 h. After the incubation period, the colonies were transferred to paper and photographed.

### Flow cytometry.

Strains carrying pFY4535 (25) were grown in gentamycin-containing tTBS medium with shaking overnight at 24°C and then subcultured for 16 to 24 h under the same conditions. An aliquot (1 μl) of each sample was added to 1 ml of phosphate-buffered saline (PBS), and these diluted samples were then evaluated for production of RFP and green fluorescent protein (GFP) using the LSRFortessa flow cytometer (BD Biosciences, San Jose, CA) using the AmCyan and PE-Texas Red channels. The data were then analyzed using FloJo software (Ashland, OR). The resulting data were first gated on live cells using forward scatter (FSC) and side scatter (SSC) and then for AmCyan + RFP double-positive cells. The geometric mean fluorescence intensities of the PE-Texas Red were quantified, analyzed, and graphed, where the y-axes were normalized to mode to account for differences in event counts of the samples.

### Transcriptome analysis.

Transcriptomes were sequenced and analyzed using modifications of previously described protocols (51). Samples were grown in shaking liquid cultures of tTBS, tTBS supplemented with 10 mM CaCl_2_, or tTBS supplemented with 10 mM CaCl_2_ and 9.7 mM pABA at 24°C for 4 to 8 h. Cells were collected by pelleting 0.5 ml of culture through centrifugation and frozen at −80°C overnight. RNA was then extracted via the MirVana kit and protocol (Thermo Fisher Scientific, Waltham, MA). Residual DNA was removed via the Turbo DNA-free kit (Invitrogen, Carlsbad, CA). Sample libraries were prepared using Tecan Genomics (NuGEN) universal total RNA seq kit and sequenced at the University of North Caroline (UNC) High-Throughput Sequencing Facility (HTSF) with the HiSeq 4000 platform (single-end 50-bp reads). Quality scores were calculated for each sequence, and low-quality sequences were removed using Trimmomatic (52), with samples having an average quality score lower than 20 across 5 bp removed. Reads were mapped to the ES114 genome using BowTie2 (53), and mapped reads were counted using HTSeq (54). Differential expression analysis was performed via DESeq2 (55). Data are displayed in a volcano plot generated by graphing the negative log_10_ *P* value (obtained from DeSeq2) and log_2_ fold change between treatments. Principal coordinate analysis (PCoA) was performed using a Bray-Curtis-based dissimilarity matrix from the vegan package in R (56). Heatmap of hierarchical clustering analysis was performed using the heatmap function in R, where transcript values were scaled for each gene.

### Statistics.

All error bars shown represent standard deviations. Prism 9 (GraphPad, San Diego, CA, USA) was used to generate graphs and perform statistical analyses. One-way or two-way analyses of variance (ANOVAs) and unpaired *t* tests were used to analyze data for each graph as noted. For ANOVAs, either Tukey’s multiple-comparisons test or Šídák’s multiple-comparison test was used, where the independent variable was on the *x* axis and the dependent variable was on the *y* axis.

### Data availability.

Data have been deposited as BioProject PRJNA759339, with BioSamples SAMN21163381 (TBS at 4 h), SAMN21163382 (TBS plus calcium at 4 h), SAMN21163383 (TBS plus Ca and PABA at 4 h), SAMN21163384 (TBS at 8 h), SAMN21163385 (TBS plus calcium at 8 h), SAMN21163386 (planktonic cells from TBS plus Ca and PABA at 8 h), and SAMN21163387 (pelleted cells from TBS plus Ca and PABA at 8 h).
