# Supplementary figures and images for: Para-Aminobenzoic Acid, Calcium, and c-di-GMP Induce Formation of Cohesive, Syp-Polysaccharide-Dependent Biofilms in Vibrio fischeri
Source: mBio. 2021 Oct 5;12(5):e02034-21. doi: 10.1128/mBio.02034-21 (PMC8546588; doi:10.1128/mBio.02034-21)

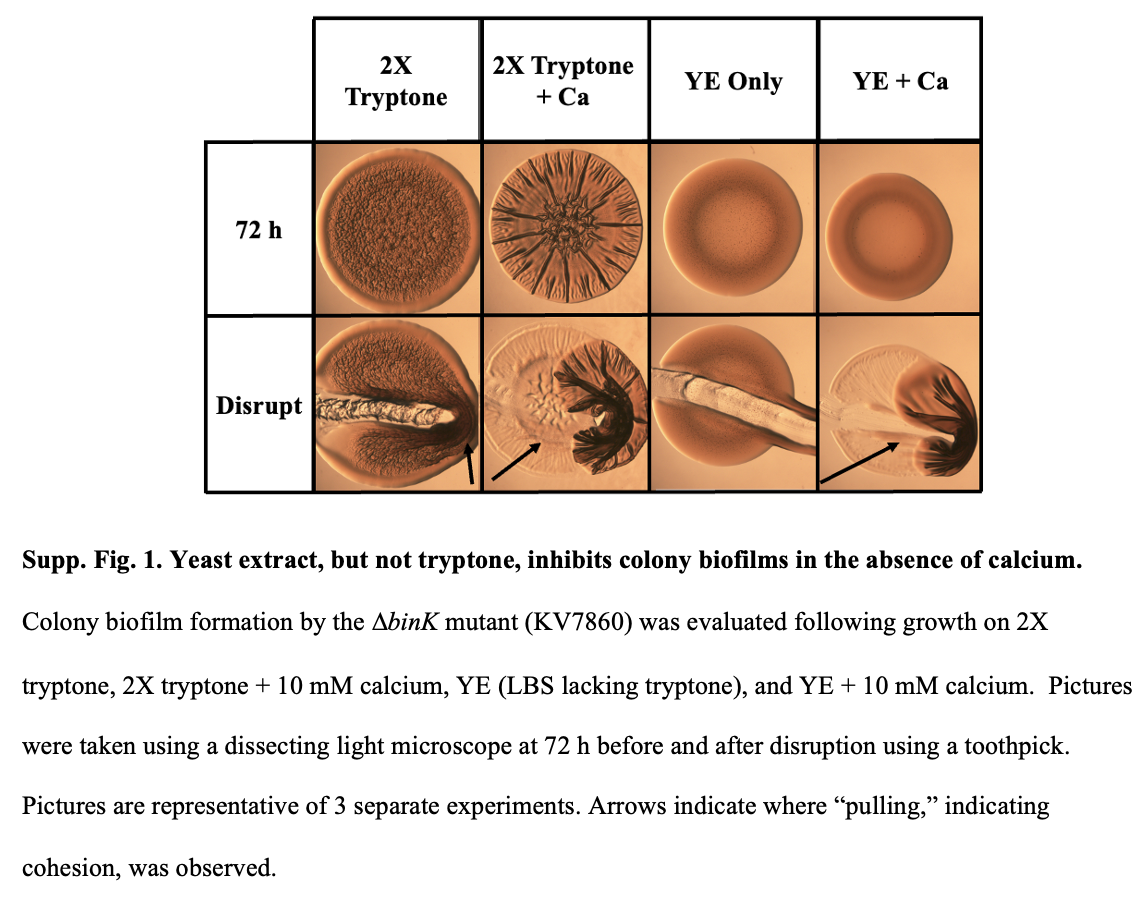

Supplement: FIG S1 [file mbio.02034-21-sf001.tif]

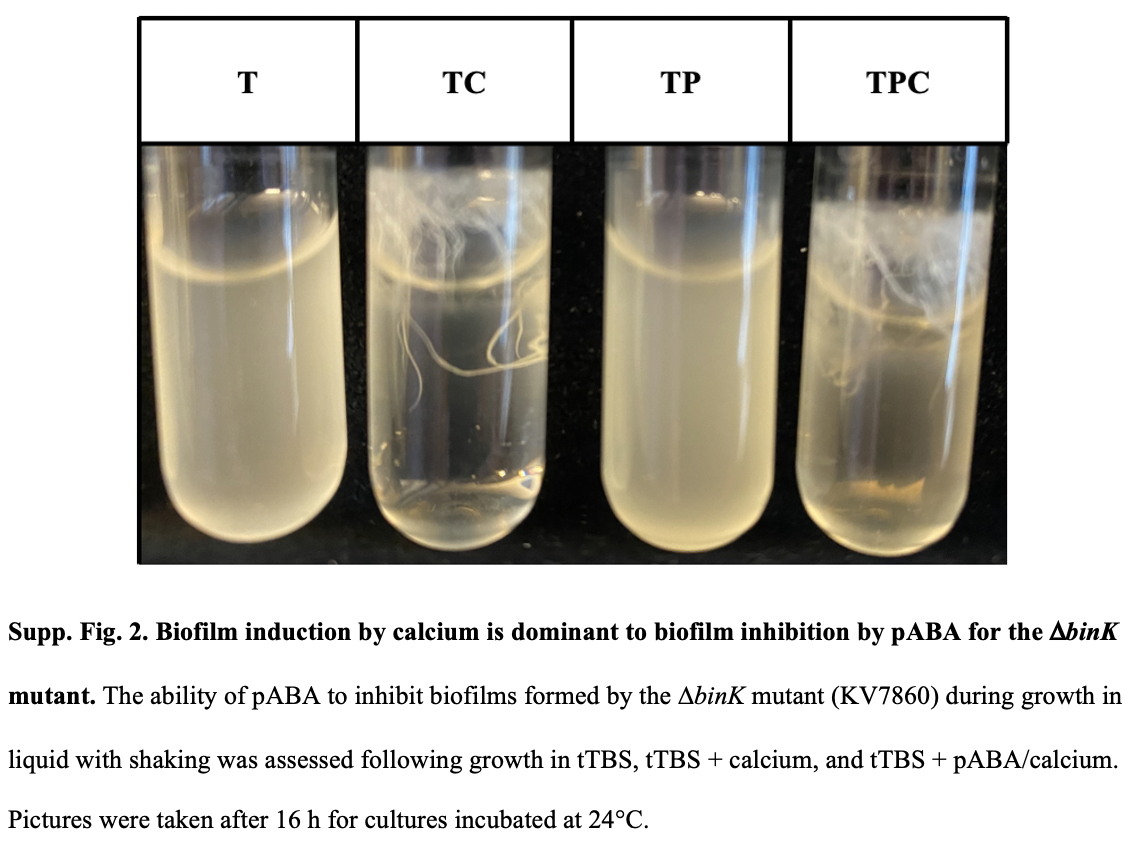

Supplement: FIG S2 [file mbio.02034-21-sf002.tif]

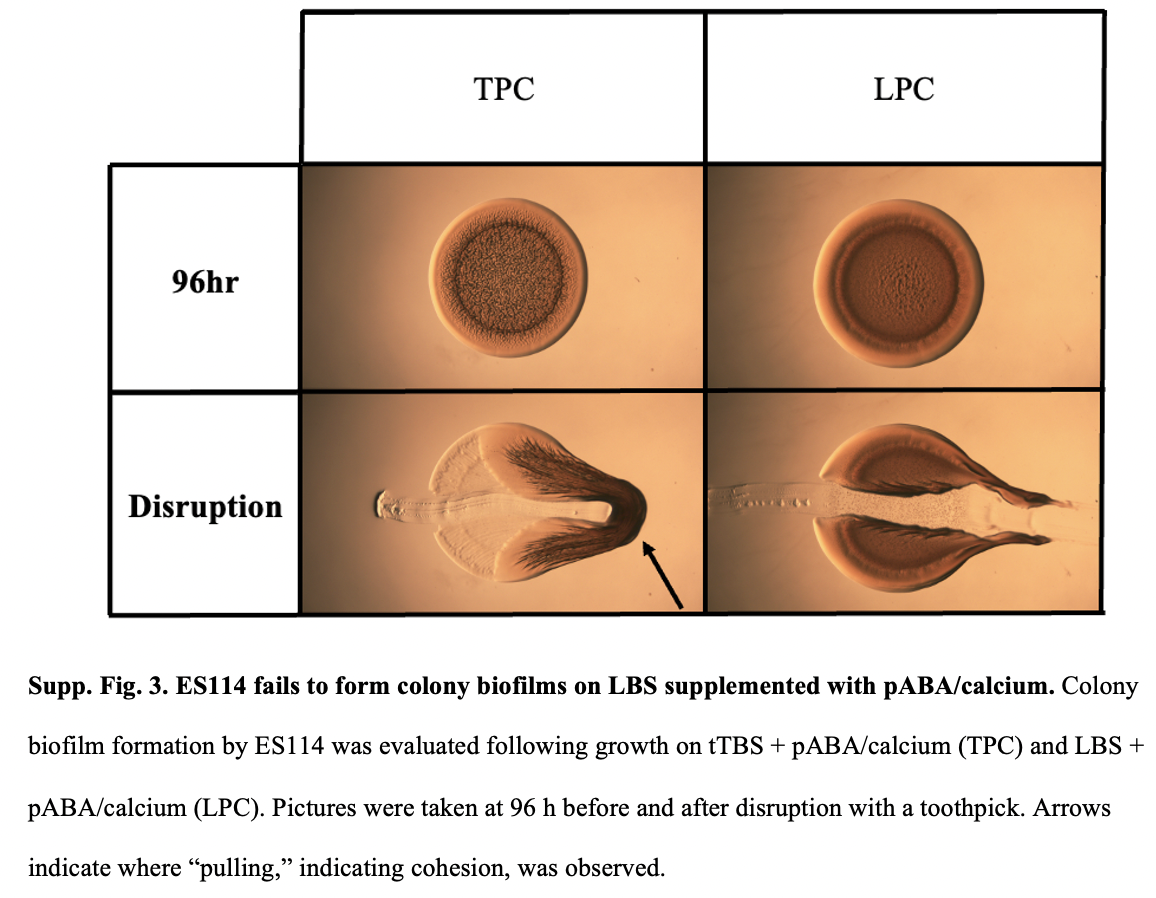

Supplement: FIG S3 [file mbio.02034-21-sf003.tif]

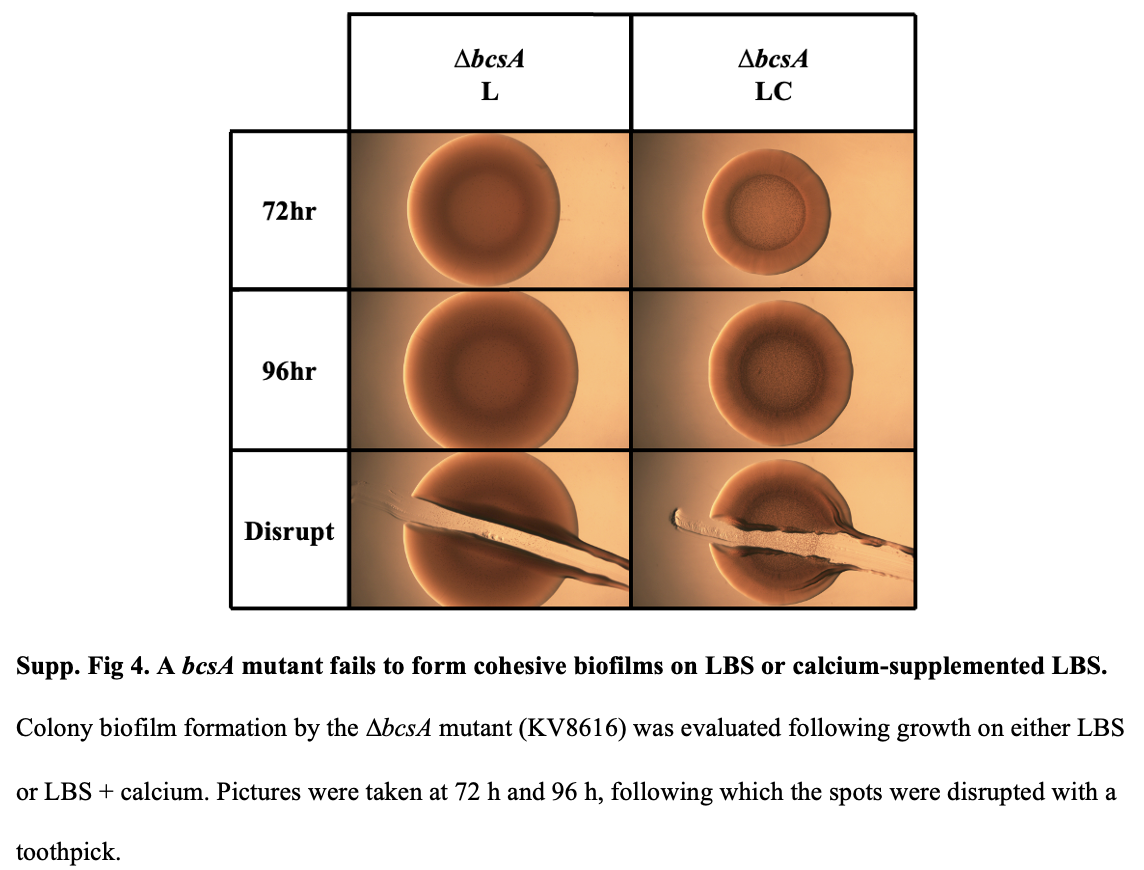

Supplement: FIG S4 [file mbio.02034-21-sf004.tif]

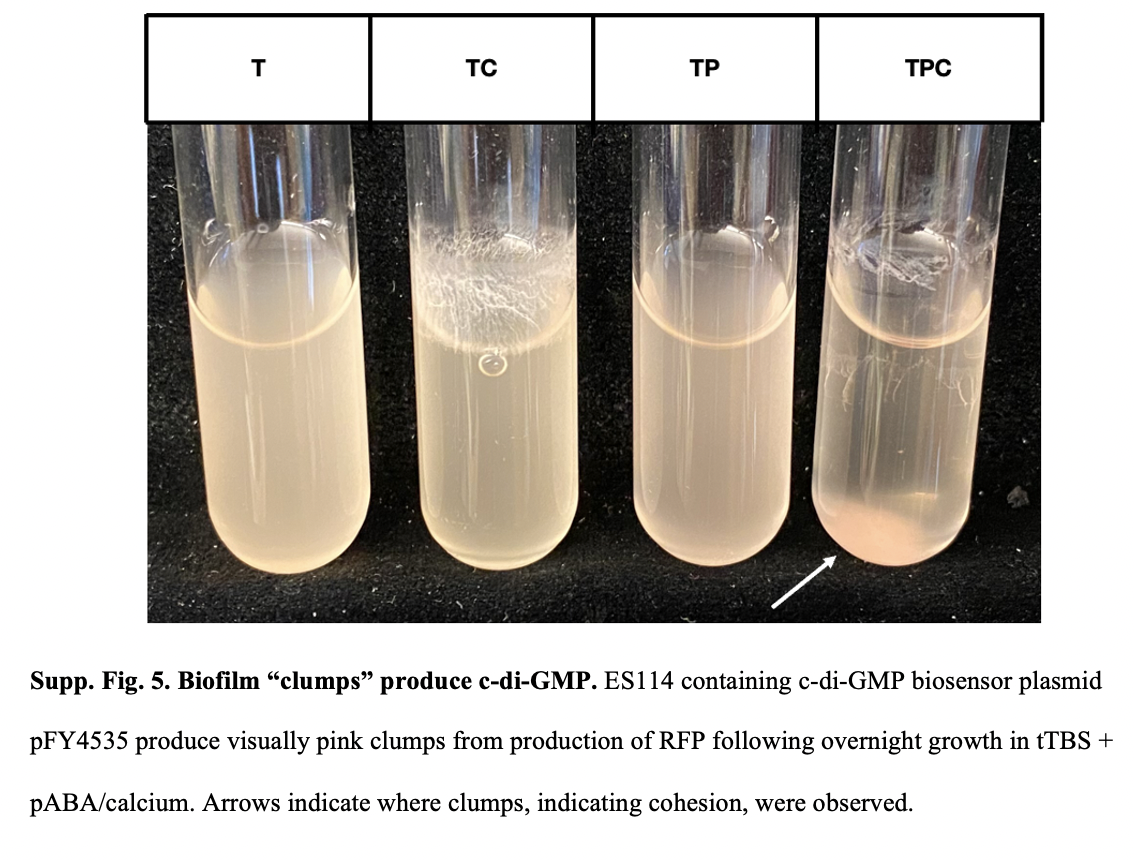

Supplement: FIG S5 [file mbio.02034-21-sf005.tif]
